# Spatial analysis identifies *LAMTOR2* overexpression in hepatocellular carcinoma with vessels encapsulating tumor clusters

**DOI:** 10.1007/s12072-026-11115-2

**Published:** 2026-06-25

**Authors:** Yoon Jung Hwang, San Ha Hwang, Min Ji Kang, Geon Woo Park, Hyeewon Seo, Hyejung Lee, Hyun Je Kim, Suk Kyun Hong, Haeryoung Kim

**Affiliations:** 1 Department of Pathology, Seoul National University College of Medicine, Seoul, Korea; 2 Department of Pathology, Seoul National University Bundang Hospital, Seongnam, Korea; 3 Department of Biomedical Sciences, Seoul National University Graduate School, Seoul, Korea; 4 Department of Microbiology and Immunology, Seoul National University College of Medicine, Seoul, Korea; 5 Cancer Research Institute, Seoul National University College of Medicine, Seoul, Korea; 6 Interdisciplinary Program in Artificial Intelligence (IPAI), Seoul National University, Seoul, Korea; 7 Genomic Medicine Institute, Medical Research Center, Seoul National University College of Medicine, Seoul, Korea; 8 Division of HBP Surgery, Department of Surgery, Seoul National University Hospital, Seoul National University College of Medicine, Seoul, Korea; 9 Department of Pathology, Seoul National University Hospital, Seoul, Korea

**Keywords:** Carcinoma, Hepatocellular, Angiogenesis, Gene expression profiling, Spatial transcriptomics, RNA-seq, *LAMTOR2*, Immunohistochemistry, Single-cell gene expression analysis, Tumor microenvironment

## Abstract

**Background:**

Vessels encapsulating tumor clusters (VETC) is a distinct angiogenic pattern in hepatocellular carcinoma (HCC). While VETC-positive HCC is associated with poor prognosis, it demonstrates improved responses to vascular-targeted therapies. However, the molecular signatures that define VETC-positive HCC remain elusive. This study employed spatial transcriptomics and single-cell RNA sequencing to characterize the molecular landscape of VETC-positive HCC and identify candidate biomarkers.

**Methods:**

Using GeoMx Digital Spatial Profiling, we analyzed tumor and adjacent liver tissues from 24 HCC patients, focusing on VETC-positive, VETC-negative, or non-neoplastic liver regions. Differential gene expression and pathway enrichment analyses were performed. Candidate genes were further evaluated using immunohistochemistry, The Cancer Genome Atlas (TCGA) dataset, and single-cell RNA sequencing.

**Results:**

Spatial transcriptomic analysis identified 39 genes upregulated in VETC-positive regions, with enrichment of angiogenesis, WNT/β-catenin, Hedgehog, and p53 signaling, epithelial–mesenchymal transition, and fatty acid metabolism pathways, and suppression of immune-related pathways. Among these, *LAMTOR2*, *DPP4*, *ZNHIT1*, and *MLXIPL* were consistently upregulated in VETC-positive regions compared to both VETC-negative and normal liver regions. *LAMTOR2* demonstrated tumor-specific localization in TCGA dataset. Immunohistochemistry confirmed that peripheral accentuation pattern of LAMTOR2 expression was restricted to tumor cells and strongly correlated with RNA expression and VETC positivity. Single-cell RNA sequencing further revealed *LAMTOR2* upregulation in malignant hepatocytes, particularly within VETC-high subpopulations enriched for lipid degradation, fatty acid oxidation, and detoxification pathways.

**Conclusion:**

VETC-positive HCC exhibits a distinct transcriptomic and metabolic profile. *LAMTOR2*, which is consistently upregulated in VETC-positive HCCs, may contribute to angiogenic and metabolic reprogramming, offering potential implications for therapeutic stratification in HCC.

**Graphical abstract:**

**Figure:**
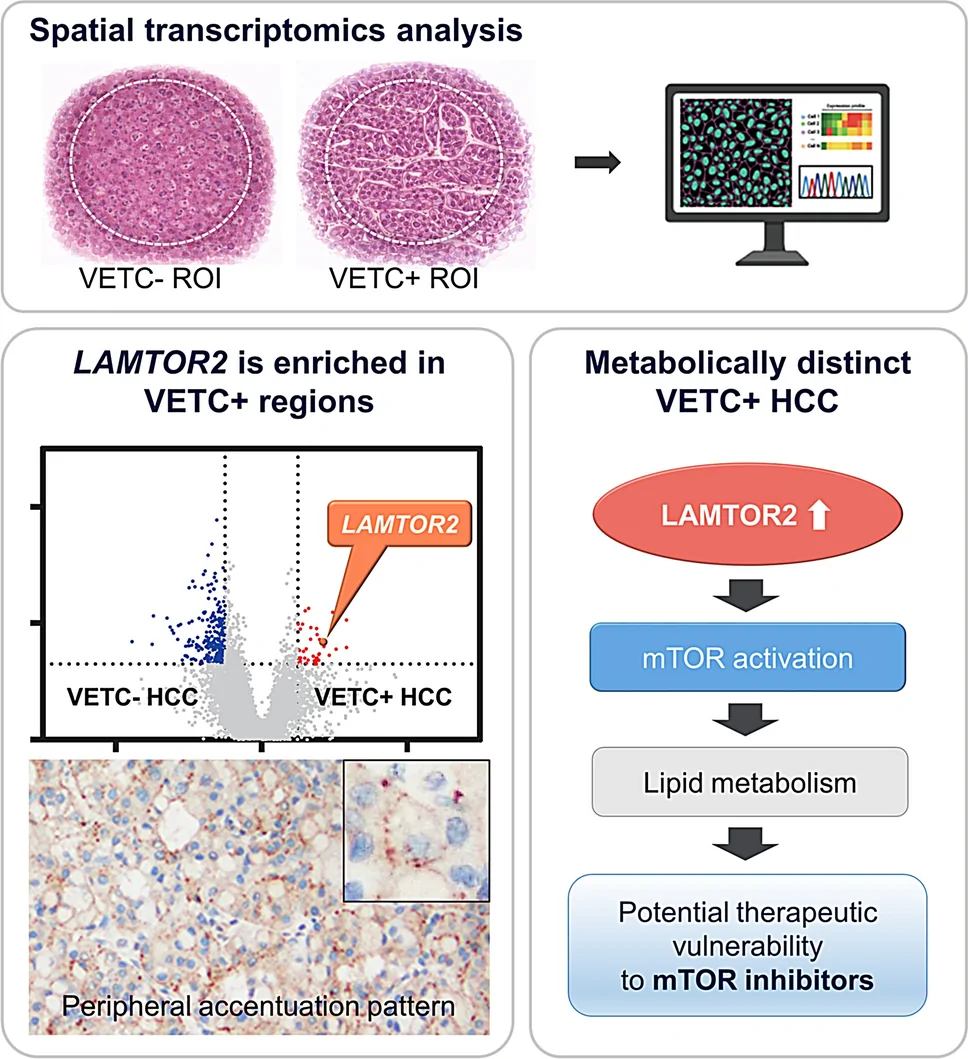

**Supplementary Information:**

The online version contains supplementary material available at 10.1007/s12072-026-11115-2.

## Introduction

Hepatocellular carcinoma (HCC) remains a major cause of cancer-related mortality, underscoring the need for improved molecular stratification [1]. The vessels encapsulating tumor clusters (VETC) pattern is a distinct angiogenic mechanism in HCC characterized by tumor cells being entirely surrounded by endothelial cells [2–5]. VETC pattern is an independent predictor of poor prognosis, associated with higher recurrence rates and lower overall survival [2–7]. On the other hand, VETC-positive HCCs have demonstrated better responses to sorafenib treatment and transarterial chemoembolization (TACE) [2, 8–10]. VETC formation is regulated by angiogenic signaling pathways, most notably angiopoietin-2, and is associated with an immunosuppressive, mTOR-activated tumor microenvironment, suggesting therapeutic potential for angiopoietin-2 inhibitors, immune checkpoint inhibitors, and mTOR inhibitors like rapamycin [2, 11–16]. Despite its clinical relevance, molecular markers that define VETC-positive HCC remain elusive.

Spatial transcriptomics offers insights into the molecular heterogeneity of HCC. It also provides a detailed view of the tumor microenvironment that is not captured by bulk RNA sequencing [18–20]. Previous studies have demonstrated that spatially distinct regions within primary liver cancers exhibit marked transcriptional and immune heterogeneity, influencing tumor behavior and therapeutic response [18, 19]. Given the prognostic and therapeutic implications of VETC pattern, comparative spatial analysis of VETC-positive and VETC-negative tumor regions may reveal molecular mechanisms underlying VETC formation and identify clinically relevant biomarkers.

In this study, we applied spatial transcriptomics to characterize gene expression differences between VETC-positive and VETC-negative regions in HCC, with the aim of identifying VETC-associated biomarkers with prognostic and therapeutic relevance.

## Materials and methods

### Patient cohorts and clinicopathological analysis

A total of 24 adult patients who underwent surgical resection for HCC at Seoul National University Hospital between September 2022 and March 2023 were prospectively enrolled. For validation, two additional retrospective cohorts were included: an immunohistochemistry (IHC) cohort of 276 resected HCC cases and a single-cell RNA sequencing (scRNA-seq) cohort of five biopsied HCC cases. This study was approved by the Institutional Review Board of Seoul National University Hospital. Clinicopathological data were obtained from medical records and pathology review. Details are provided in the supplementary methods.

### Spatial transcriptomics

Tumor and adjacent non-neoplastic liver tissues were collected during surgical resection, formalin-fixed, paraffin-embedded, and used to construct 2-mm core tissue microarrays (TMAs). After histologic review, representative tumor and non-neoplastic regions were selected for analysis.

**Fig. 1:**
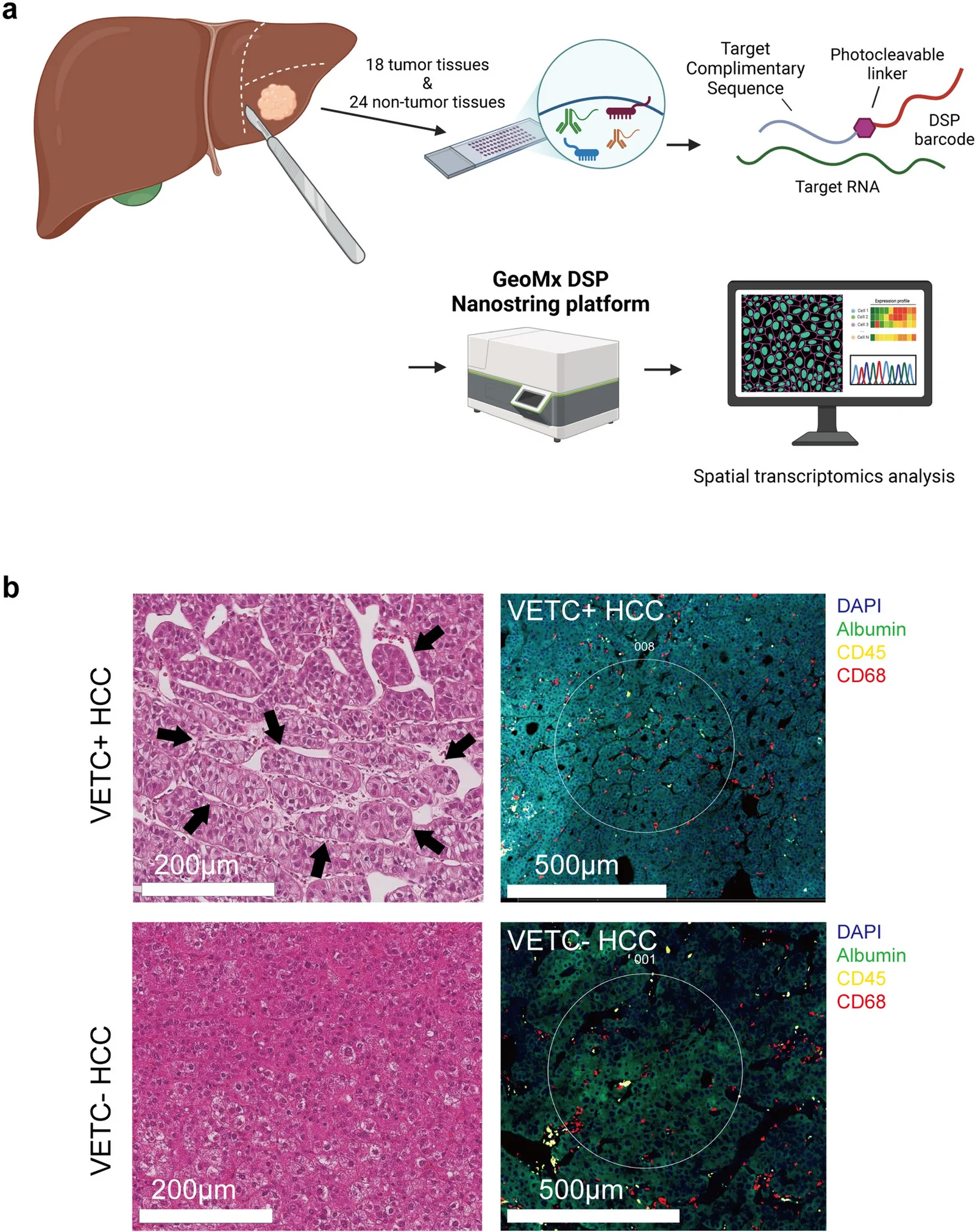
Spatial transcriptomics workflow. **a** Schematic representation of the study workflow. Tumor (*n* = 18) and non-tumor (*n* = 24) liver tissue samples were collected from hepatocellular carcinoma (*HCC*) patients undergoing surgical resection. Tissue samples were processed and analyzed using the GeoMx Digital Spatial Profiler (*DSP*) Nanostring platform. Target RNA molecules were hybridized with complementary sequences conjugated to a DSP barcode linked by a photo-cleavable linker. Following cleavage and collection, spatial transcriptomic analysis was conducted. **b** Representative hematoxylin and eosin (*HE*) and immunofluorescence images of selected regions of interest (*ROIs*) within HCC tissues. Left: Representative HE images showing vessels encapsulating tumor clusters (*VETC*)-positive and VETC-negative HCC (100x, scale bars: 200 µm. Arrows indicate VETC). Right: Corresponding multiplexed immunofluorescence images for DAPI (blue), Albumin (green), CD45 (cyan), and CD68 (red). White circles indicate the ROIs selected for GeoMx DSP analysis. (*DSP* Digital Spatial Profiler, *VETC + HCC* vessels encapsulating tumor clusters-positive hepatocellular carcinoma, *VETC−HCC* vessels encapsulating tumor clusters-negative hepatocellular carcinoma)

Spatial gene expression profiling was conducted on TMA slides using the GeoMX Digital Spatial Profiler (NanoString Technologies, Inc., Seattle, WA, USA) (Fig. 1a). Regions of interest (ROIs) were selected based on fluorescent imaging overlaid on hematoxylin and eosin (HE)-stained images. Tumor regions were classified as VETC-positive (presence of any VETC focus) or VETC-negative, based on hematoxylin–eosin (HE) stains and CD34 immunohistochemistry (Fig. 1b and Supplementary Fig. 1). The presence of any VETC focus was regarded as VETC-positive. A total of 42 ROIs, including seven VETC-positive, 11 VETC-negative, and 24 non-neoplastic liver regions, were selected. Libraries were sequenced using the NovaSeq 6000 system (Illumina, Inc., San Diego, CA, USA), and processed via the GeoMx NGS pipeline. Differential expression and pathway analyses were performed using the GeoMx DSP Analysis Suite (details in supplementary methods).

### Immunohistochemistry

Immunohistochemistry (IHC) for LAMTOR2 and CD34 was performed (see supplementary methods and Supplementary Fig. 2).

### Single-cell RNA sequencing

Single-cell RNA sequencing (scRNA-seq) was performed on five HCC cases [21]. Data were processed using Seurat, malignant hepatocytes were identified using CopyKAT, and VETC-related signatures were analyzed (see supplementary methods).

### Statistical analysis

Statistical analysis was performed using commercially available software (RStudio software for Windows Version: 2022.12.0 + 353; Graph Pad Prism software Version 10; Python version 3.11.8). Continuous data were presented as median with interquartile range, and were evaluated using Student’s t test, Mann–Whitney test, and Kruskal–Wallis test. Categorical variables were analyzed by chi-square test and Fisher’s exact test. *p* Value of < 0.05 was considered statistically significant.

## Results

### Baseline characteristics

A total of 18 patients with HCC were included in this study. Baseline characteristics showed no significant differences in age, tumor size, or other clinicopathological variables between the two groups based on the presence or absence of the VETC pattern (Supplementary Table 1).

### Digital spatial profiling

#### Global transcriptomic differences

**Fig. 2:**
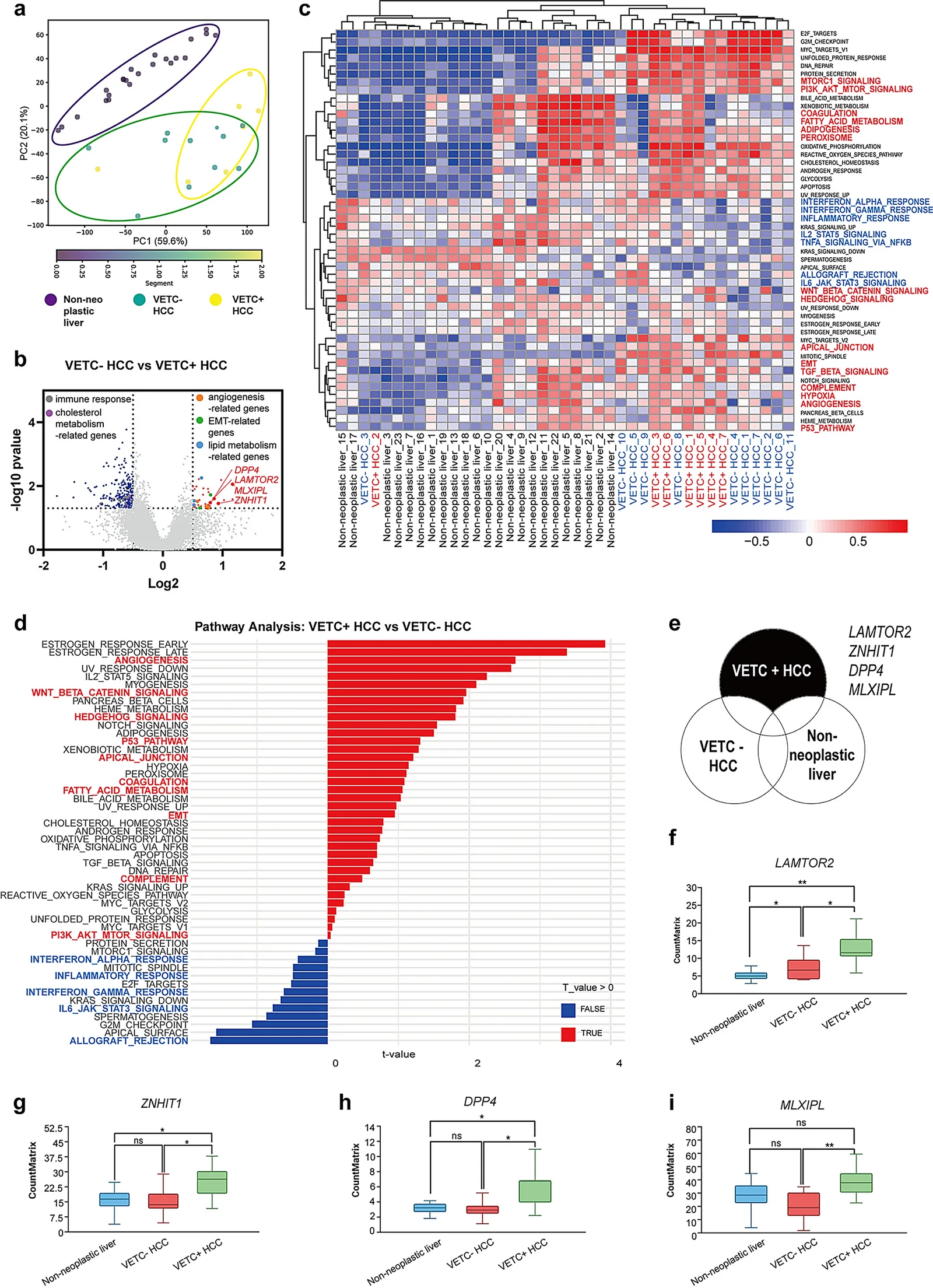
Digital spatial profiling. **a** Principal component analysis (*PCA*) plot based on gene expression profiles. The non-neoplastic liver tissue (purple) clusters in the upper right region, clearly distinguishing itself from the tumor samples. Vessels encapsulating tumor clusters (*VETC*)-positive hepatocellular carcinoma (*HCC*) (teal) and VETC-negative HCC (yellow) partially overlap but also form distinct clusters in some areas. **b** Volcano plot comparing differentially expressed genes (*DEGs*) between VETC-positive and VETC-negative HCC. Genes significantly upregulated in VETC-positive HCC include *LAMTOR2*, *ZNHIT1*, *DPP4*, and *MLXIPL* (red dots). Angiogenesis-related genes (orange dots), epithelial–mesenchymal transition (EMT)-related genes (blue dots) and lipid metabolism-related genes (green dots) are upregulated in VETC-positive HCC. Immune-related genes (grey dots) and cholesterol metabolism-related genes (purple dots) are downregulated in VETC-positive HCC. **c** Heatmap of hallmark pathway enrichment scores in HCC and non-neoplastic liver tissues. In VETC-positive tumors, pathways related to mTOR, lipid metabolism, WNT/β-catenin, Hedgehog, EMT, and angiogenesis are enriched, while pathways related to inflammation are suppressed. Rows represent hallmark gene sets, and columns correspond to individual samples. The samples are categorized into VETC-positive HCC (red labels), VETC-negative HCC (blue labels), and non-neoplastic liver tissue (black labels). The color scale represents pathway enrichment scores, with red indicating upregulation and blue indicating downregulation of the respective pathways. Hierarchical clustering is applied to both samples and pathways. **d** Quantification of gene set variation analysis using VETC-positive HCC-specific DEGs. Enrichment of angiogenesis, WNT/β-catenin, Hedgehog, EMT, p53 signaling, and lipid metabolism pathways is observed in VETC-positive tumors, with concomitant suppression of interferon- and inflammatory signaling pathways. Red bars indicate pathways enriched in VETC-positive HCC, while blue bars represent pathways enriched in VETC-negative HCC. **e** Venn diagram showing consistent upregulation of four genes (*LAMTOR2*, *ZNHIT1*, *DPP4*, and *MLXIPL*) in VETC-positive HCC compared to VETC-negative HCC and non-neoplastic liver tissues. Among the upregulated genes, four genes—*LAMTOR2*, *DPP4*, *ZNHIT1* and *MLXIPL*—were consistently increased in VETC-positive regions compared with both VETC-negative tumors and non-neoplastic liver tissue. **f**–**i** Boxplots of expression levels of key DEGs (*LAMTOR2*, *ZNHIT1*, *DPP4*, and *MLXIPL*) across VETC-positive HCC, VETC-negative HCC, and non-neoplastic liver tissues. **f** *LAMTOR2* expression was significantly higher in VETC-positive HCC compared to both VETC-negative HCC and non-neoplastic tissue. *LAMTOR2* expression was also elevated in VETC-negative HCC compared to non-neoplastic liver tissue. **g** *ZNHIT1* expression was significantly increased in VETC-positive HCC compared to both VETC-negative HCC and non-neoplastic tissue, with no significant difference between VETC-negative HCC and non-neoplastic liver tissue. **h** *DPP4* expression was significantly higher in VETC-positive HCC compared to VETC-negative HCC and non-neoplastic tissue, but no significant difference was observed between VETC-negative HCC and non-neoplastic liver tissue. **i** *MLXIPL* expression was increased in VETC-positive HCC compared to VETC-negative HCC, but no significant difference was found between VETC-positive and VETC-negative HCC and non-neoplastic liver tissue. Statistical significance is indicated by *p < 0.05 and **p < 0.01. "ns" denotes non-significant differences. (*VETC−HCC* vessels encapsulating tumor clusters-negative hepatocellular carcinoma, *VETC + HCC* vessels encapsulating tumor clusters-positive hepatocellular carcinoma, *EMT* epithelial mesenchymal transition)

Principal component analysis (PCA) demonstrated clear separation between tumor and non-neoplastic liver samples, with partial distinction between VETC-positive and VETC-negative HCC (Fig. 2a).

#### Differential gene expression

Spatial transcriptomic analysis identified differentially expressed genes (DEGs) between VETC-positive and VETC-negative regions, and DEGs between VETC-positive regions and normal liver tissue (Fig. 2b and Supplementary Fig. 3a–c). In VETC-positive HCC, 39 upregulated and 235 downregulated DEGs were identified compared to VETC-negative HCC. The upregulated genes were involved in angiogenesis, epithelial–mesenchymal transition (EMT), and lipid metabolism. Conversely, the downregulated genes were associated with immune response and cholesterol metabolism.

#### Pathway enrichment analysis

Gene set variation analysis (GSVA) revealed consistent enrichment of angiogenesis, WNT/β-catenin, Hedgehog, EMT, p53 signaling, and lipid metabolism pathways in VETC-positive tumors, with concomitant suppression of interferon and inflammatory signaling pathways (Fig. 2c, d, Supplementary Fig. 6c).

#### Identification of candidate genes

Among the upregulated genes, four genes—*LAMTOR2*, *DPP4*, *ZNHIT1* and *MLXIPL*—were consistently increased in VETC-positive regions compared with both VETC-negative tumors and non-neoplastic liver tissue (Fig. 2e–i). All four genes exceeded the limit of quantitation (LOQ) value in almost all areas of interest (Supplementary Fig. 3d). These findings suggest that these genes may serve as key molecular markers of VETC-positive HCC, warranting further investigation.

### Immunohistochemical validation

**Fig. 3:**
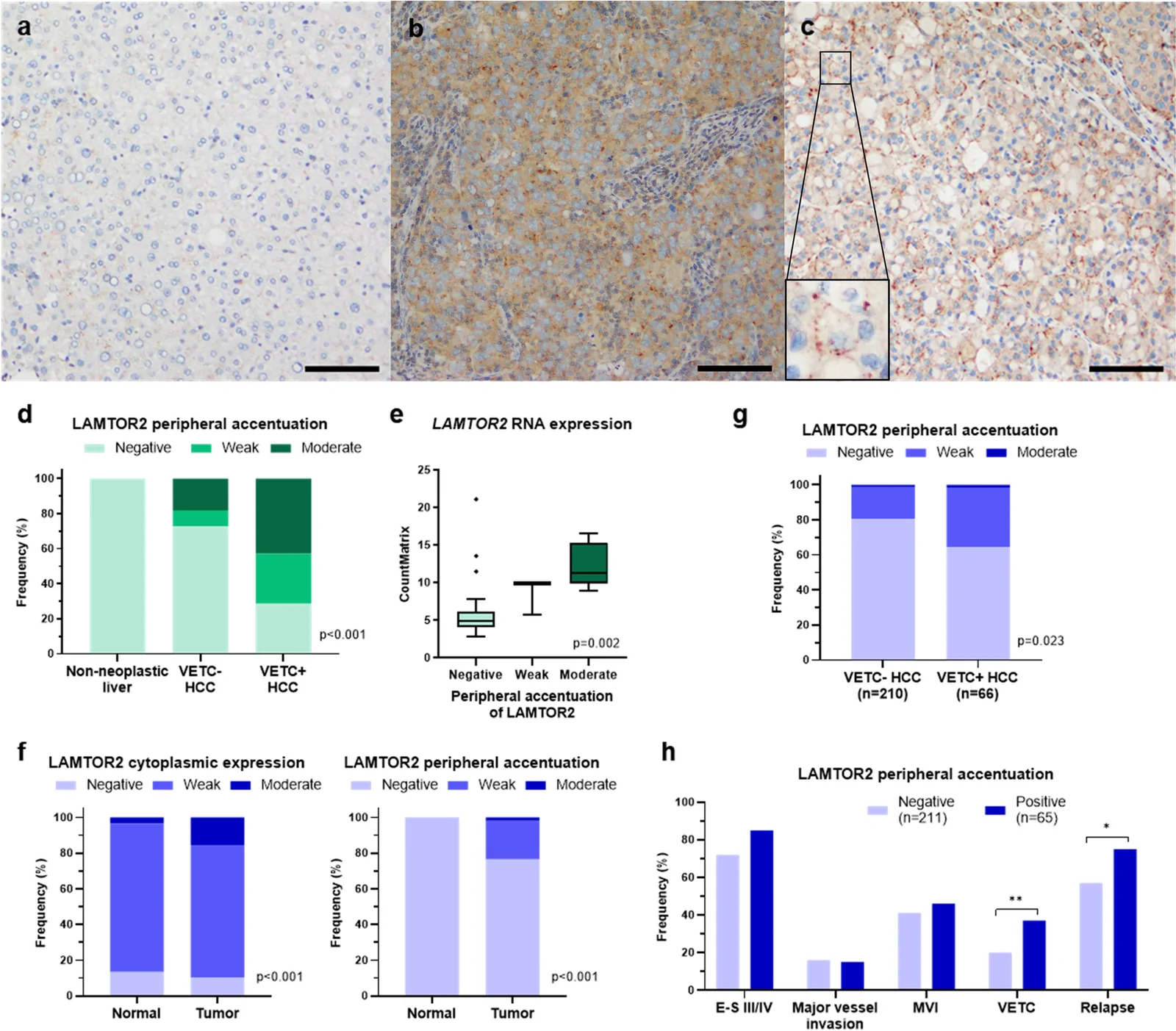
Immunohistochemical analysis of LAMTOR2 expression. **a**–**c** Representative immunohistochemistry (IHC) images of LAMTOR2 expression (200x, scale bars: 100 µm). **a** A non-neoplastic liver tissue showing focal faint or no cytoplasmic expression in hepatocytes. **b** Vessels encapsulating tumor clusters (VETC)-negative hepatocellular carcinoma (HCC) displaying moderate cytoplasmic expression in tumor cells without cell peripheral accentuation. **c** VETC-positive HCC showing a peripheral accentuation pattern of expression with moderate intensity in tumor cells. **d**, **e** Analysis of LAMTOR2 IHC expression and correlation with RNA levels in the study cohort (*n* = 42). **d** Distribution of peripheral accentuation intensity levels in non-neoplastic liver, VETC-negative HCC, and VETC-positive HCC. The frequency of peripheral accentuation pattern was higher in VETC-positive HCC compared to VETC-negative HCC, and moderate intensity of peripheral accentuation was more common in VETC-positive HCC as well. No peripheral accentuation pattern was observed in non-neoplastic liver. Chi-square test showed a significant *p* value between the three groups. **e** A linear correlation between LAMTOR2 peripheral accentuation intensity and RNA expression levels, indicating that higher peripheral staining is associated with increased RNA expression, with a significant *p* value observed in the Kruskal–Wallis test. **f**–**h** Validation of LAMTOR2 IHC findings in the independent tumor microarray (*TMA*)-based HCC cohort (*n* = 276). **f** Comparison of LAMTOR2 cytoplasmic expression and peripheral accentuation intensity between tumor cores (*n* = 579) and normal cores (*n* = 289). Both cytoplasmic expression and peripheral accentuation pattern were observed at higher frequencies in tumor tissue compared to normal tissue. The peripheral accentuation pattern was exclusive to tumor tissue. **g** Comparison of LAMTOR2 peripheral accentuation intensity between VETC-positive HCC (*n* = 66) and VETC-negative HCC (*n* = 210). The frequency of peripheral accentuation pattern was higher in VETC-positive HCC compared to VETC-negative HCC, and moderate intensity was also more common in VETC-positive HCC. **h** Clinicopathological findings, including Edmondson–Steiner (E–S) grade, vascular invasion, VETC status and tumor relapse, according to the presence of LAMTOR2 peripheral accentuation pattern. Statistical significance is indicated as *p < 0.05 and **p < 0.01. (*VETC−HCC* vessels encapsulating tumor clusters-negative hepatocellular carcinoma, *VETC + HCC*, vessels encapsulating tumor clusters-positive hepatocellular carcinoma, *E–S* Edmondson–Steiner grade, *MVI* microvascular invasion, *VETC* vessels encapsulating tumor clusters)

Based on The Cancer Genome Atlas (TCGA) data, we observed that *LAMTOR2* RNA expression was significantly elevated in tumor tissues compared to normal tissues (Supplementary Results and Supplementary Fig. 4). Consequently, we decided to evaluate LAMTOR2 protein expression through IHC staining performed on TMA slides for further validation. IHC revealed distinct expression patterns of LAMTOR2 across normal liver tissue, VETC-negative HCC, and VETC-positive HCC. In normal liver tissue, LAMTOR2 showed either negative staining or faint to weak cytoplasmic expression in hepatocytes, with no peripheral accentuation (Fig. 3a). In HCC, LAMTOR2 was expressed in the cytoplasm of tumor cells, and a subset of cases demonstrated peripheral accentuation, a pattern exclusive to HCC (Fig. 3b, c). This peripheral accentuation pattern was significantly more frequent in VETC-positive HCC (*n* = 5/7, 71%) compared to VETC-negative HCC (*n* = 3/11, 27%) or non-neoplastic liver tissue (*n* = 0/24, *p* < 0.001). In addition, moderate intensity of peripheral accentuation was observed at a significantly higher rate in VETC-positive HCC (*n* = 3/7, 43%) compared to VETC-negative HCC (*n* = 2/11, 18%) or non-neoplastic liver tissue (*n* = 0/24, *p* < 0.001, Fig. 3d).

A strong statistical correlation was observed between peripheral accentuation pattern of LAMTOR2 and its RNA expression levels (median (IQR) RNA expression with and without peripheral accentuation; 10.4 (9.6–12.0) vs. 5.0 (4.1–6.1); *p* < 0.001). Furthermore, a linear relationship was identified between the intensity of peripheral accentuation and RNA expression levels (negative: 5.0 (4.1–6.1), weak: 9.9 (7.8–9.9), moderate: 11.3 (10.9–13.9), *p* = 0.002, Fig. 3e). These findings demonstrate that LAMTOR2 protein localization and intensity, particularly peripheral accentuation, are closely linked to its RNA expression.

### LAMTOR2 protein expression in a validation cohort

LAMTOR2 IHC staining was performed on the IHC validation cohort of 276 HCC cases. For each case, 1–4 tumor cores and 1–2 normal cores were included, resulting in 579 tumor cores and 289 normal cores analyzed. There was significant difference in LAMTOR2 expression between tumor and normal tissues. Among tumor cores, 16% (90/579) exhibited moderate cytoplasmic expression, 74% (429/579) weak expression, and 10% (60/579) negative expression, compared to 3% (10/289), 83% (240/289), and 13% (39/289) in normal cores, respectively (*p* < 0.001, Fig. 3f). Notably, peripheral accentuation of LAMTOR2, a feature exclusive to tumor tissue, was observed in 24% (136/579) of cases. Specifically, 2% (11/579) of tumor cores showed moderate expression, and 22% (125/579) weak expression, whereas no peripheral accentuation was detected in normal tissue cores (*p* < 0.001).

Of the 276 cases analyzed, 65 cases (24%) exhibited LAMTOR2 peripheral accentuation. HCCs with peripheral accentuation demonstrated a significant association with VETC positivity, compared to cases without peripheral accentuation (24/65, 37% vs. 42/211, 20%; *p* = 0.008, Fig. 3g, h and Supplementary Table 2). Furthermore, these cases exhibited a marginally higher proportion of Edmondson–Steiner grade III/IV tumors (55/65, 83% vs. 152/211, 72%; *p* = 0.060) and a significantly higher recurrence rate (49/65, 75% vs. 120/211, 57%; *p* = 0.011). No significant differences were observed in the frequency of vascular invasion or the macrotrabecular massive (MTM) subtype between cases with and without peripheral accentuation. Although microvascular invasion (MVI) and MTM subtype have been previously reported to associate with the VETC pattern, and were also more frequent in VETC-positive tumors in our cohort (MVI: 39/66, 59% vs. 77/210, 37%; *p* = 0.002; MTM: 12/66, 20% vs. 11/210, 10%; *p* = 0.092), their distribution did not significantly differ according to LAMTOR2 expression. These findings highlight the unique expression patterns of LAMTOR2 in HCC, particularly its peripheral localization, which is strongly associated with VETC positivity and aggressive tumor features.

### Single-cell transcriptomic analysis

**Fig. 4:**
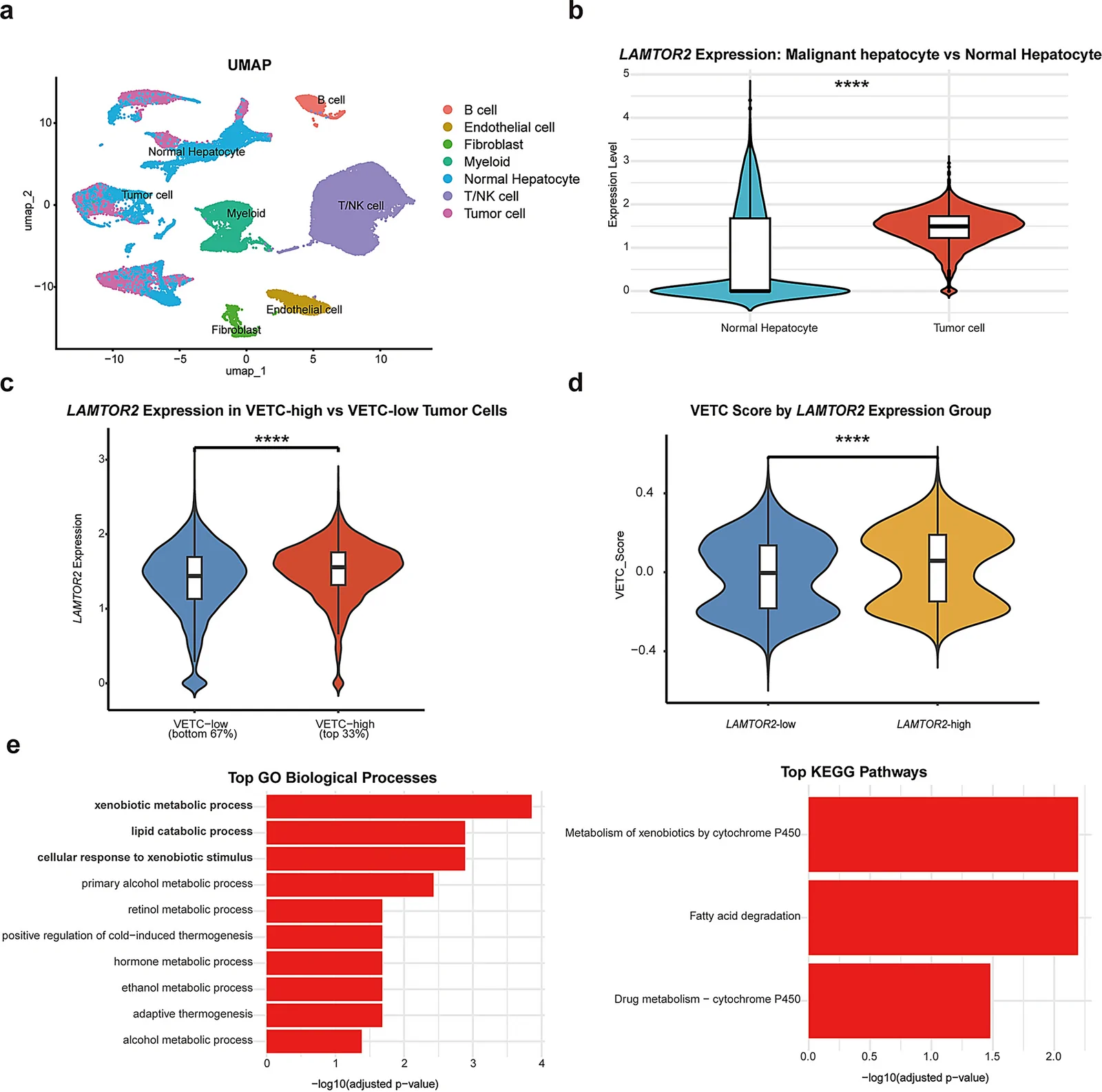
Single-cell RNA sequencing. **a** Uniform Manifold Approximation and Projection (UMAP) visualization of single-cell RNA-sequencing data from five hepatocellular carcinoma (*HCC*) patients, showing clustering of seven major cell types. **b** *LAMTOR2* expression is significantly higher in copyKAT-defined aneuploid HCC cells compared to diploid non-neoplastic hepatocytes (**** indicates *p* < 0.0001). **c** Violin plot of *LAMTOR2* expression in tumor cells stratified by VETC score-based grouping (VETC-high = top 33% vs VETC-low = bottom 67%; *n* = 12,247 tumor cells from five HCC patients, including 362 CopyKAT-confirmed malignant cells from HCC 02 [VETC-positive]). VETC-high tumor cells exhibited significantly elevated *LAMTOR2* expression compared with VETC-low cells (**** indicates *p* < 0.0001). **d** Violin plot showing that VETC module scores are significantly higher in *LAMTOR2*-high tumor cells compared with *LAMTOR2*-low tumor cells (**** indicates *p* < 0.0001), supporting the reciprocal direction of the VETC–*LAMTOR2* association observed in (**c**). **e** Gene ontology (GO, left) and Kyoto Encyclopedia of Genes and Genomes (KEGG, right) pathway enrichment plots of differentially expressed genes from VETC-high, *LAMTOR2*-high tumor cells exhibit significant upregulation of pathways including fatty acid degradation, lipid catabolic process, metabolism of xenobiotics by cytochrome P450, and drug metabolism–cytochrome P450. (*UMAP* Uniform Manifold Approximation and Projection, *VETC + HCC* vessels encapsulating tumor clusters-positive hepatocellular carcinoma, *VETC* vessels encapsulating tumor clusters)

To further substantiate these observations, scRNA-seq was performed on the scRNA-seq validation cohort of five biopsied HCC cases, including one VETC-positive case. Basic information is presented in Supplementary Table 3, and detailed results are provided in supplementary results section. Unsupervised clustering identified seven major cell clusters (Fig. 4a, Supplementary Figs. 5a, d, 6g), among which *LAMTOR2* expression was markedly elevated in HCC cells compared to other cell types (*p* < 0.0001, Fig. 4b, Supplementary Fig. 5b, c).

To further dissect the molecular phenotype of VETC-positive HCC, we applied a scoring approach based on DEGs identified from spatial transcriptomic analysis of VETC-positive versus VETC-negative HCC regions (log₂ fold change > 0.75, Supplementary Fig. 6a, b). Uniform Manifold Approximation and Projection (UMAP) embedding of HCC cells followed by VETC signature scoring revealed clear stratification of tumor cells into VETC-high and VETC-low subpopulations (Supplementary Fig. 5e), which recapitulated gene expression patterns observed in the spatial transcriptomics data (Supplementary Fig. 5f–h).

*LAMTOR2* expression was significantly higher in the VETC-high HCC group compared to the VETC-low group (*p* < 0.0001, Fig. 4c), further supporting its association with this vascular phenotype. Additionally, cells with high *LAMTOR2* expression exhibited significantly elevated VETC scores compared to the *LAMTOR2*-low group (*p* < 0.0001, Figs. 4d, Supplementary Fig. 6d–f), further confirming this association.

Subsequently, we isolated VETC-high HCC cells with high *LAMTOR2* expression and performed differential gene expression and pathway enrichment analysis. This revealed significant upregulation of pathways associated with lipid degradation and detoxification processes, including fatty acid degradation, lipid catabolic process, metabolism of xenobiotics by cytochrome P450, and drug metabolism–cytochrome P450 (Fig. 4e).

## Discussion

The VETC pattern is a unique form of angiogenesis in HCC, where endothelial cells entirely enclose tumor clusters [2]. Clinically, VETC-positive HCC has two key implications: first, it is associated with higher metastatic potential, and second, it shows enhanced response to vascular-targeted therapies [2–6, 8–10]. This paradox underscores the need to elucidate the molecular mechanisms underlying VETC-positive HCC.

In this study, we applied GeoMx Digital spatial profiling and scRNA-seq to characterize the molecular landscape of VETC-positive HCC. Spatial analysis revealed that VETC-positive tumors exhibit a distinct transcriptional profile. Importantly, hallmark pathways related to angiogenesis, WNT/β-catenin signaling, Hedgehog signaling, EMT, p53 signaling, and lipid metabolism were enriched in VETC-positive tumors. These findings suggest that VETC-positive HCCs rely on vascular remodeling, enhanced invasiveness, and metabolic reprogramming. In contrast, immune-related pathways were suppressed, indicating an immunosuppressive tumor microenvironment. This transcriptomic pattern is further supported by an independent immunohistochemical cohort (*n* = 229) showing significantly reduced CD8 + T cell and CD163 + macrophage densities in the tumor center of VETC-positive HCC [22], supporting the interpretation that down-regulated immune-related pathways in VETC-positive tumors reflect altered immune cell infiltration rather than a tumor-intrinsic property. This transcriptomic observation is biologically concordant with the recent demonstration that VETC-positive HCC harbors an immunosuppressive microenvironment enriched in TNFRSF4 + regulatory T cells and exhausted PD1 + CD8 + T cells driven by endothelial TGF-β1 signaling [17], and provides a complementary spatial transcriptomic perspective on the tumor compartment of this niche.

Among DEGs, four candidates—*LAMTOR2*, *MLXIPL*, *ZNHIT1*, and *DPP4*—were consistently upregulated in VETC-positive HCC compared to both VETC-negative HCC and non-tumorous liver tissue. However, only *LAMTOR2* showed significantly elevated expression in HCC relative to matched normal liver tissues in the TCGA cohort.

Given this consistent and tumor-specific overexpression, *LAMTOR2* emerged as a central marker in our study. Spatial transcriptomic analysis showed its significant overexpression in VETC-positive tumors, and this was validated by IHC, which revealed distinct peripheral accentuation of LAMTOR2 in tumor cells, especially in the VETC-positive subgroup. This staining pattern was exclusive to tumor. Moreover, LAMTOR2 peripheral expression intensity correlated strongly with RNA expression level.

To further investigate the biological significance of *LAMTOR2*, we conducted scRNA-seq, which revealed that *LAMTOR2* expression was significantly upregulated in malignant hepatocytes, particularly within a VETC-high subpopulation defined by DEGs derived from spatial transcriptomics data. VETC-high and *LAMTOR2*-high tumor cells demonstrated enrichment in pathways related to lipid degradation and cytochrome P450-mediated detoxification. Integrating these results with spatial transcriptomic data, which showed upregulation of both lipid synthesis and fatty acid oxidation pathways, suggests that VETC-positive HCCs exhibit dual metabolic programs. Lipid synthesis likely supports structural demands and cell growth, whereas lipid degradation and oxidation may provide energy and substrates to sustain vascular remodeling. This metabolic flexibility may enable VETC-positive tumors to maintain their distinctive vascular structure and support tumor progression within a relatively hypoxic microenvironment.

Collectively, these findings indicate that *LAMTOR2* is potentially a functional mediator of the vascular and metabolic phenotype observed in VETC-positive HCC. *LAMTOR2* (late endosomal/lysosomal adaptor, MAPK and mTOR activator 2) is a component of the Regulator/LAMTOR complex, which is a scaffold protein complex that anchors multiprotein signaling units on late endosomes/lysosomes, thereby activating the MAPK and mTOR pathways [23, 24]. Both pathways have been strongly implicated in HCC tumorigenesis, angiogenesis, and metabolic activity [25, 26]. Findings are consistent with prior reports linking mTOR activity to VETC formation, supporting the hypothesis that *LAMTOR2* may act upstream or synergistically with mTOR in shaping the VETC phenotype. Interestingly, a pro-tumorigenic role for LAMTOR2 has been demonstrated in pancreatic ductal adenocarcinoma, where VSIG2 recruits LAMTOR2 to mTOR, thereby promoting mTOR phosphorylation and correlating with poor survival [27]. This cross-cancer mechanistic evidence supports the rationale for considering LAMTOR2 as a prognosis-associated and therapeutically actionable target in VETC-positive HCC. This raises the possibility of *LAMTOR2* as a predictive marker for response to mTOR inhibitors, such as rapamycin. Combined with recent evidence that Treg depletion restores anti-tumor immunity in VETC-positive HCC [17], our findings further support the rationale for combining mTOR-pathway inhibition with immune checkpoint blockade as a stratified therapeutic strategy in this subset. Notably, LAMTOR2 expression did not significantly correlate with vascular invasion, suggesting that its role in tumor progression may be independent of conventional invasion mechanisms. Further studies are warranted to investigate the functional role of *LAMTOR2* in VETC formation and its potential as a therapeutic target.

The peripheral accentuation pattern of *LAMTOR2* expression on immunohistochemistry demonstrated positive correlation with VETC status; however, this was only seen in 36% of VETC-positive tumors in our 276-case validation cohort (Fig. 3g and Supplementary Table 2), limiting its potential use as a biomarker in diagnostic practice. However, *LAMTOR2* may be explored as a predictive marker for response to mTOR inhibitors in VETC-positive HCC, and also as a prognostic marker—patients with LAMTOR2 peripheral accentuation in our cohort exhibited a significantly higher postoperative recurrence rate. Recent deep-learning radiopathomics and MRI-based models have shown that VETC can be predicted preoperatively from imaging and histopathology [28, 29], establishing VETC as a prognostically critical pathological feature deserving non-invasive prediction tools; our identification of LAMTOR2 as a molecularly-defined VETC-associated marker complements these imaging-based approaches by providing a tissue-level biomarker for VETC biology characterization and therapeutic stratification. Future research should focus on validating the correlation between LAMTOR2 expression and long-term clinical outcomes, including recurrence-free and overall survival, in larger multicenter cohorts.

A key limitation of this study is the relatively small sample size, which may limit the robustness of molecular stratification. scRNA-seq cohort included only a single VETC-positive patient and publicly available HCC scRNA-seq datasets do not provide direct VETC pathological annotation. To mitigate these limitations, we performed a thorough re-examination of the single VETC-positive patient (HCC 02) in our in-house dataset and reframed the analysis at the single-cell level. We further corroborated the protein-level findings in an independent IHC cohort of 276 HCC cases. Nevertheless, larger multicenter cohorts with paired VETC histopathological annotation will be required to firmly establish the generalizability of our observations.

Another limitation is related to tumor heterogeneity. First, while we aimed to analyze VETC-specific transcriptomes by selecting VETC areas through GeoMx, the ROIs we selected contained some non-VETC regions, which may have contributed to the overlap between VETC-positive and VETC-negative HCC. Indeed, VETC in HCC appears to exist along a continuous spectrum, ranging from tumors with only focal or mosaic VETC to those with a more diffuse distribution of VETC [30]. Therefore, rather than complete clustering separation, a certain degree of overlap accompanied by directional trends may more accurately reflect the biological reality of clinical tumor samples. Second, LAMTOR2 staining was analyzed exclusively in TMA cores, which represent only a part of the tumor, and do not provide information about the overall expression pattern of LAMTOR2 throughout the entire tumor. Third, the scRNA-seq analysis integrated data from five heterogeneous HCC samples, and the VETC-high transcriptional signature was based on DEGs, not the exact definition of VETC. As such, it may not directly correlate with VETC status. Consequently, further studies analyzing whole tumor sections with more detailed VETC classification, as well as larger and more homogeneous cohorts, would be necessary to comprehensively evaluate whether LAMTOR2 expression is uniform or localized to specific regions, such as VETC-positive areas. This is an important consideration for understanding the full extent of LAMTOR2’s expression in HCC and its potential as a biomarker for VETC.

Additionally, while our study establishes a strong association between *LAMTOR2* and VETC-positive HCC, it does not provide direct functional validation of *LAMTOR2*’s role in VETC formation and tumor progression. Further in vitro and in vivo studies are required to determine the relationship between *LAMTOR2* expression and VETC-driven tumor biology. Finally, future research on the transcriptomics of endothelial cells in VETC-positive and VETC-negative regions could provide valuable insights into the molecular mechanisms underlying VETC formation.

In conclusion, our study provides novel insights into the molecular landscape of VETC-positive HCC. Moreover, we identify *LAMTOR2* as a potential supportive, prognosis-associated marker and a potential therapeutic target for VETC-positive HCC. Future studies should further investigate the mechanistic role of *LAMTOR2* in VETC formation and metabolic adaptations in VETC-positive HCC, as well as explore the therapeutic potential of targeting *LAMTOR2*.

## Supplementary Information

Below is the link to the electronic supplementary material.

Supplementary file1 (DOCX 71393 kb)

## Data Availability

All data generated or analyzed during this study are included in this article and its supplementary material files. Further inquiries can be directed to the corresponding author.

## Abbreviations

DEG: Differentially expressed gene
EMT: Epithelial–mesenchymal transition
HCC: Hepatocellular carcinoma
IHC: Immunohistochemistry
MTM: Macrotrabecular massive
MVI: Microvascular invasion
ROI: Regions of interest
scRNA-seq: Single-cell RNA sequencing
TCGA: The Cancer Genome Atlas
TMA: Tissue microarray
VETC: Vessels encapsulating tumor clusters

## References

[1] Bray F, Laversanne M, Sung H, Ferlay J, Siegel RL, Soerjomataram I, et al. Global cancer statistics 2022: GLOBOCAN estimates of incidence and mortality worldwide for 36 cancers in 185 countries. CA Cancer J Clin. 2024;74(3):229–263 38572751 10.3322/caac.21834

[2] Liu K, Dennis C, Prince DS, Marsh-Wakefield F, Santhakumar C, Gamble JR, et al. Vessels that encapsulate tumour clusters vascular pattern in hepatocellular carcinoma. JHEP Rep. 2023;5(8): 100792 37456680 10.1016/j.jhepr.2023.100792 PMC10339254

[3] Huang CW, Lin SE, Huang SF, Yu MC, Tang JH, Tsai CN, et al. The vessels that encapsulate tumor clusters (VETC) pattern is a poor prognosis factor in patients with hepatocellular carcinoma: an analysis of microvessel density. Cancers (Basel). 2022;14(21):5428 36358846 10.3390/cancers14215428 PMC9658947

[4] Kawasaki J, Toshima T, Yoshizumi T, Itoh S, Mano Y, Wang H, et al. Prognostic impact of vessels that encapsulate tumor cluster (VETC) in Patients who underwent liver transplantation for hepatocellular carcinoma. Ann Surg Oncol. 2021;28(13):8186–8195 34091774 10.1245/s10434-021-10209-5

[5] Renne SL, Woo HY, Allegra S, Rudini N, Yano H, Donadon M, et al. Vessels encapsulating tumor clusters (VETC) Is a powerful predictor of aggressive hepatocellular carcinoma. Hepatology. 2020;71(1):183–195 31206715 10.1002/hep.30814

[6] Dennis C, Prince DS, Moayed-Alaei L, Remash D, Carr-Boyd E, Bowen DG, et al. Association between vessels that encapsulate tumour clusters vascular pattern and hepatocellular carcinoma recurrence following liver transplantation. Front Oncol. 2022;12: 997093 36387254 10.3389/fonc.2022.997093 PMC9643778

[7] Guan R, Lin W, Zou J, Mei J, Wen Y, Lu L, et al. Development and validation of a novel nomogram for predicting vessels that encapsulate tumor cluster in hepatocellular carcinoma. Cancer Control. 2022;29:10732748221102820 35609265 10.1177/10732748221102820 PMC9136459

[8] Lin C, He Y, Liu M, Wu A, Zhang J, Li S, et al. Vessels that encapsulate tumor clusters (VETC) predict cTACE response in hepatocellular carcinoma. J Hepatocell Carcinoma. 2023;10:383–397 36915392 10.2147/JHC.S395903 PMC10007987

[9] Lin W, Lu L, Zheng R, Yuan S, Li S, Ling Y, et al. Vessels encapsulating tumor clusters: a novel efficacy predictor of hepatic arterial infusion chemotherapy in unresectable hepatocellular carcinoma. J Cancer Res Clin Oncol. 2023;149(19):17231–17239 37801135 10.1007/s00432-023-05444-0 PMC11798139

[10] Fang JH, Xu L, Shang LR, Pan CZ, Ding J, Tang YQ, et al. Vessels that encapsulate tumor clusters (VETC) Pattern is a predictor of sorafenib benefit in patients with hepatocellular carcinoma. Hepatology. 2019;70(3):824–839 30506570 10.1002/hep.30366

[11] Shigematsu Y, Tanaka K, Amori G, Kanda H, Takahashi Y, Takazawa Y, et al. Potential involvement of oncostatin M in the immunosuppressive tumor immune microenvironment in hepatocellular carcinoma with vessels encapsulating tumor clusters. Hepatol Res. 2024;54(4):368–381 37950386 10.1111/hepr.13988

[12] Toshida K, Itoh S, Toshima T, Yoshiya S, Goto R, Mita A, et al. Clinical significance of mechanistic target of rapamycin expression in vessels that encapsulate tumor cluster-positive hepatocellular carcinoma patients who have undergone living donor liver transplantation. Ann Gastroenterol Surg. 2024;8(1):163–171 38250695 10.1002/ags3.12735 PMC10797838

[13] Zhang P, Ono A, Fujii Y, Hayes CN, Tamura Y, Miura R, et al. The presence of vessels encapsulating tumor clusters is associated with an immunosuppressive tumor microenvironment in hepatocellular carcinoma. Int J Cancer. 2022;151(12):2278–2290 36054900 10.1002/ijc.34247

[14] Akiba J, Nakayama M, Kondo R, Kusano H, Ogasawara S, Mihara Y, et al. Immunophenotypes and tumor immune microenvironment in hepatocellular carcinoma with macrotrabecular massive and vessels encapsulating tumor clusters. In Vivo. 2024;38(2):640–646 38418151 10.21873/invivo.13483 PMC10905475

[15] Itoh S, Yoshizumi T, Yugawa K, Imai D, Yoshiya S, Takeishi K, et al. Impact of immune response on outcomes in hepatocellular carcinoma: association with vascular formation. Hepatology. 2020;72(6):1987–1999 32112577 10.1002/hep.31206

[16] Kurebayashi Y, Ojima H, Tsujikawa H, Kubota N, Maehara J, Abe Y, et al. Landscape of immune microenvironment in hepatocellular carcinoma and its additional impact on histological and molecular classification. Hepatology. 2018;68(3):1025–1041 29603348 10.1002/hep.29904

[17] Huang BY, Mi ZQ, Zhang XY, Ji YC, Wu MZ, Cheng ZF, et al. Vessels encapsulating tumor clusters promote noninvasive metastasis of hepatocellular carcinoma by shaping an immunosuppressive microenvironment. J Clin Invest. 2026. 10.1172/JCI193758 41493808 10.1172/JCI193758 PMC12904702

[18] Wu R, Guo W, Qiu X, Wang S, Sui C, Lian Q, et al. Comprehensive analysis of spatial architecture in primary liver cancer. Sci Adv. 2021;7(51):eabg3750 34919432 10.1126/sciadv.abg3750 PMC8683021

[19] Zhao N, Zhang Y, Cheng R, Zhang D, Li F, Guo Y, et al. Spatial maps of hepatocellular carcinoma transcriptomes highlight an unexplored landscape of heterogeneity and a novel gene signature for survival. Cancer Cell Int. 2022;22(1):57 35109839 10.1186/s12935-021-02430-9 PMC8812006

[20] Zhou PY, Zhou C, Gan W, Tang Z, Sun BY, Huang JL, et al. Single-cell and spatial architecture of primary liver cancer. Commun Biol. 2023;6(1):1181 37985711 10.1038/s42003-023-05455-0 PMC10661180

[21] Park GW, Jang H, Nguyen-Phuong T, Nam H, Park C-G, Choi GH, et al. Integrative single-cell and spatial transcriptomic analysis reveals MAIT cell dysfunction in relapsed HCC. J Hepatocell Carcinoma. 2026;13:1–17 10.2147/JHC.S575861 PMC13008124 41878224

[22] Taniai T, Haruki K, Vayrynen JP, Gomisawa K, Sato S, Furukawa K, et al. Immunological and prognostic significance of vessels encapsulating tumor clusters (VETC) in hepatocellular carcinoma. Eur J Surg Oncol. 2025;51(10):110381 40818297 10.1016/j.ejso.2025.110381

[23] Lamberti G, De Smet CH, Angelova M, Kremser L, Taub N, Herrmann C, et al. LAMTOR/Ragulator regulates lipid metabolism in macrophages and foam cell differentiation. FEBS Lett. 2020;594(1):31–42 31423582 10.1002/1873-3468.13579 PMC7003824

[24] Scheffler JM, Sparber F, Tripp CH, Herrmann C, Humenberger A, Blitz J, et al. LAMTOR2 regulates dendritic cell homeostasis through FLT3-dependent mTOR signalling. Nat Commun. 2014;5:5138 25336251 10.1038/ncomms6138 PMC4220488

[25] Ferrin G, Guerrero M, Amado V, Rodriguez-Peralvarez M, De la Mata M. Activation of mTOR signaling pathway in hepatocellular carcinoma. Int J Mol Sci. 2020;21(4):1266 32070029 10.3390/ijms21041266 PMC7072933

[26] Moon H, Ro SW. MAPK/ERK signaling pathway in hepatocellular carcinoma. Cancers (Basel). 2021;13(12):3026 34204242 10.3390/cancers13123026 PMC8234271

[27] Xu J, Quan G, Huang W, Jiang J. VSIG2 promotes malignant progression of pancreatic ductal adenocarcinoma by enhancing LAMTOR2-mediated mTOR activation. Cell Commun Signal. 2023;21(1):223 37626304 10.1186/s12964-023-01209-x PMC10463957

[28] Yu Y, Cao L, Shen B, Du M, Gu W, Gu C, et al. Deep learning radiopathomics models based on contrast-enhanced MRI and pathologic imaging for predicting vessels encapsulating tumor clusters and prognosis in hepatocellular carcinoma. Radiol Imaging Cancer. 2025;7(2): e240213 40084948 10.1148/rycan.240213 PMC11966553

[29] Zhang C, Zhong H, Zhao M, Li Y, Dai Z, Pang G. Delta radiomics-based nomogram for preoperative prediction vessels encapsulating tumor clusters (VETC) and prognosis in hepatocellular carcinoma using dynamic contrast-enhanced CT. BMC Med Imaging. 2026. 10.1186/s12880-026-02210-9 41761127 10.1186/s12880-026-02210-9 PMC13040871

[30] Zhu Y, Wang M, Cao J, Song H, Zhang P. Vessels encapsulating tumor clusters in hepatocellular carcinoma: a distinct metastatic pathway with diagnostic and therapeutic significance. J Transl Med. 2026;24(1):185 41555423 10.1186/s12967-025-07354-w PMC12896066

