# Supplementary material for: Spatial analysis identifies LAMTOR2 overexpression in hepatocellular carcinoma with vessels encapsulating tumor clusters
Source: Hepatol Int. 2026 Jun 25;20(4):1150–61. doi: 10.1007/s12072-026-11115-2 (PMC13518457; doi:10.1007/s12072-026-11115-2)
Supplement: Supplementary file 1 [file 12072_2026_11115_MOESM1_ESM.docx]

**Spatial Analysis Identifies *LAMTOR2* Overexpression in Hepatocellular Carcinoma with Vessels Encapsulating Tumor Clusters**

Yoon Jung Hwang^1,2*^, San Ha Hwang^3,4,5*^, Min Ji Kang^3,4^, Geon Woo Park^3,4,5^, Hyeewon Seo^3,4,5^, Hyejung Lee^1^, Hyun Je Kim^3,4,5,6,7^, Suk Kyun Hong^8^, Haeryoung Kim^1,9^

^1^Department of Pathology, Seoul National University College of Medicine, Seoul, Korea

^2^Department of Pathology, Seoul National University Bundang Hospital, Seongnam, Korea

^3^Department of Biomedical Sciences, Seoul National University Graduate School, Seoul, Korea

^4^Department of Microbiology and Immunology, Seoul National University College of Medicine, Seoul, Korea

^5^Cancer Research Institute, Seoul National University College of Medicine, Seoul, Korea

^6^Interdisciplinary Program in Artificial Intelligence (IPAI), Seoul National University, Seoul, Korea

^7^Genomic Medicine Institute, Medical Research center, Seoul National University College of Medicine, Seoul, Korea

^8^Department of Surgery, Seoul National University Hospital, Seoul National University College of Medicine, Seoul, Korea

^9^Department of Pathology, Seoul National University Hospital, Seoul, Korea

*Yoon Jung Hwang and San Ha Hwang equally contributed as first authors.

Corresponding authors:

Hyun Je Kim M.D., Ph.D.

Department of Biomedical Sciences, Seoul National University Graduate School

Department of Microbiology and Immunology, Seoul National University College of Medicine

Cancer Research Institute, Seoul National University College of Medicine

Interdisciplinary Program in Artificial Intelligence (IPAI), Seoul National University

Genomic Medicine Institute, Medical Research center, Seoul National University College of Medicine

103 Daehak-no, Jongno-gu, Seoul, 03080, Korea

Telephone: 82-10-4189-7756

E-mail: [tte9801@snu.ac.kr](mailto:tte9801@snu.ac.kr)

Orcid ID: 0000-0003-4467-0949

Suk Kyun Hong M.D., Ph.D.

Division of HBP surgery, Department of Surgery, Seoul National University Hospital

101, Daehak-ro, Jongno-gu, Seoul, 03080, Korea

Telephone: 82-10-9117-0831

E-mail: nobel1210@naver.com

Orcid ID: 0000-0002-0020-6215

Haeryoung Kim M.D., Ph.D.

Department of Pathology, Seoul National University College of Medicine

103 Daehak-no, Jongno-gu, Seoul, 03080, Korea

Telephone: 82-2-740-8322; Fax: 82-2-765-5600

E-mail: [haeryoung.kim@snu.ac.kr](mailto:haeryoung.kim@snu.ac.kr)

Orcid ID: 0000-0002-4205-9081

**Supplementary INFORMATION**

**Supplementary Methods**

**1. Patient Cohorts and Clinicopathological Analysis**

A total of 24 adult patients who underwent surgical resection for histologically confirmed HCC at Seoul National University Hospital between September 2022 and March 2023 were prospectively enrolled. Patients with concurrent primary malignancies of other organs or those receiving immunosuppressive therapy were excluded. Written informed consent was obtained from all participants.

For validation, two additional retrospective cohorts were included: (1) an IHC cohort comprising 276 surgically resected HCC cases from 2009 to 2011, and (2) a single-cell RNA sequencing (scRNA seq) cohort of five biopsied HCC cases obtained between 2022 and 2023. For these two cohorts, the requirement for written informed consent was waived by the Institutional Review Board due to the retrospective nature. This study was approved by the Institutional Review Board of Seoul National University Hospital (IRB Nos. H-2207-142-1342 and B-2112-727-302). All procedures were performed in compliance with relevant guidelines and regulations, adhering to the ethical principles outlined in the Declaration of Helsinki and Good Clinical Practice.

Clinical data, including patient age, sex and preoperative laboratory findings, were retrieved from electronic medical records. Pathology reports and microscopy slides (glass or digitally scanned) for all cases were reviewed by two pathologists (Y.J.H. and H.K). Tumor-related pathological parameters such as tumor size, histologic subtype, histological differentiation (Edmondson-Steiner grade), tumor stage and the presence of microvascular invasion (MVI) were recorded.

**2. Spatial Transcriptomics**

**2.1. Specimen Acquisition and Tissue Microarray Construction**

Tissue slabs smaller than 1x1x1 cm, comprising tumor, tumor-normal border, or non-tumorous liver tissue, were collected during surgery for research purposes. Specimens required for clinical diagnostics were excluded. From each patient, 3 to 4 tissue slabs were obtained, processed into formalin-fixed and paraffin-embedded (FFPE) blocks, and corresponding hematoxylin and eosin (HE)-stained slides were prepared. After histological evaluation, blocks containing significant necrosis were excluded, resulting in a total of 72 blocks from 24 patients.

Tumor and non-neoplastic liver tissue samples from these 24 HCC patients were used to construct 2 mm core tissue microarrays (TMAs). Two TMA slides were generated: one composed of 18 tumor cores, excluding 6 cases where tumor cores were unavailable, and the other of 24 non-neoplastic liver cores. These TMAs were subsequently used for both digital spatial profiling and IHC analyses.

**2.2. Digital Spatial Profiling**

Spatial gene expression profiling was conducted on TMA slides using the GeoMX Digital Spatial Profiler (NanoString Technologies, Inc., Seattle, WA, USA), according to the manufacturer’s protocols (MAN-10150, MAN-10152, MAN-10153, MAN-10154, MAN-10130-07) (**Fig. 1a**). Visualization was performed using nuclear counterstaining (SYTO13) and antibodies against albumin (clone 6B11, AF532, MA1-20127, Invitrogen), CD45 (clone D9M8I, PE, Cat #14579, Cell Signaling Technology), and CD68 (clone KP1, AF647, sc-20060AF647, Santa Cruz Biotechnology), conjugated with fluorophores using the A20182 AF532 conjugation kit. Regions of interest (ROIs) were selected based on fluorescent imaging overlaid on hematoxylin and eosin (HE)-stained images. Within the tumor cores, VETC-positive and VETC-negative regions were designated based on HE and immunohistochemistry (IHC) for CD34, under the supervision of a pathologist (Y.J.H) (**Fig. 1b and Supplementary Fig. 1**). Areas with even a single VETC focus were designated as VETC-positive regions, and cases with VETC-positive areas were classified as VETC-positive HCC. Total 42 ROIs, including seven VETC-positive, 11 VETC-negative, and 24 non-neoplastic liver regions, were selected.

Target RNA molecules were exposed by treating sections with proteinase K, followed by hybridization with UV-cleavable oligonucleotide probes targeting 18,000 RNA transcripts from the GeoMx Human Whole Transcriptome Atlas. Oligonucleotides from the ROIs were UV-cleaved, collected, and processed into sequencing libraries. Libraries were sequenced using the NovaSeq 6000 system (Illumina, Inc., San Diego, CA, USA), and raw data were converted into digital count conversion (DCC) files via the GeoMx NGS pipeline.

**2.3. Transcriptomic Data Analysis**

Data processing, including normalization, differential expression analysis, and visualization, was performed using the GeoMx DSP Analysis Suite (v3.1.0.221, GEOMX-0094). Normalization was conducted using third-quartile (Q3) normalization based on housekeeping genes to standardize gene expression data and ensure comparability across samples. Quality control procedures included 10% limit of quantification (LOQ) filtering and background noise reduction.

Differentially expressed genes (DEGs) were identified from selected ROIs annotated as VETC-positive, VETC-negative, or non-neoplastic liver tissue. Statistical comparisons were performed using Welch’s t-test, and genes with a p-value < 0.05 and an absolute log_2_ fold change > 0.5 were considered significantly differentially expressed. For pathway analysis, this threshold was used to capture broader pathway-level transcriptional alterations. The log_2_ fold change threshold of 0.5 was chosen to capture a broader range of DEGs, ensuring inclusion of potentially relevant genes. When applying a threshold of 1, only five genes were upregulated in VETC-positive HCC compared to VETC-negative HCC, and only one gene did not overlap with non-neoplastic liver tissue. However, using the 0.5 threshold, we identified four genes that were upregulated in VETC-positive HCC compared to both VETC-negative HCC and non-neoplastic liver tissue, with all showing log_2_ fold changes greater than 0.75 (**Supplementary Fig.6b**). Therefore, for subsequent scRNA seq analysis, a more specific subset of DEGs with log_2_ fold change > 0.75 was selected to define the VETC-associated signature gene set (the specific genes are listed in **Supplementary Table 4**).

Principal component analysis (PCA) was performed to visualize global transcriptomic differences based on gene expression profiles, with the first two principal components (PC1 and PC2) capturing the major variance between VETC-positive HCC, VETC-negative HCC, and non-neoplastic liver. To investigate the functional consequences of transcriptional changes, gene set variation analysis (GSVA) was conducted using the GSVA R package with hallmark gene sets obtained from the Molecular Signatures Database (MSigDB, category “H”). DEGs were used as input for GSVA to score pathway-level activity across samples. Pathways enriched in VETC-positive or VETC-negative HCC were further identified using statistical comparison of GSVA scores, including t-tests and one-way analysis of variance (ANOVA), and were visualized using heatmap and barplot.

**3. Immunohistochemistry**

IHC for LAMTOR2 and CD34 was performed. LAMTOR2 IHC was conducted on TMAs from both the prospective cohort and the retrospective IHC validation cohorts using a primary antibody against LAMTOR2 (HPA004126, 1:50 dilution; Sigma-Aldrich, St. Louis, MO, USA). CD34 IHC was performed on TMAs from the prospective cohort and tissue from the scRNA seq cohort using a primary antibody against CD34 (M716501-2; Agilent, Santa Clara, CA, USA). LAMTOR2 staining patterns were classified by intensity as negative, weak, or moderate, and by the location of expression as cytoplasmic (with or without peripheral accentuation) or peripheral (cytoplasmic expression with peripheral accentuation) (**Supplementary Fig. 2**).  The IHC slides were reviewed independently by two pathologists (Y.J.H and H.K), who were blinded to the clinical information and VETC status of the samples.

**4. Single-cell RNA sequencing**

**4.1. Gene Expression Quantification and Quality Control in Single-cell Data Processing**

Single-cell RNA sequencing (scRNA-seq) was performed on tumor specimens from five HCC patients^22^. Single-cell RNA-seq data were preprocessed using the Seurat R package (version 5.1.0.). Raw gene expression matrices were normalized and log-transformed using NormalizeData, and low-quality cells were filtered based on thresholds for mitochondrial gene content, total RNA counts, and feature numbers. Highly variable genes (HVGs) were identified using the FindVariableFeatures function with the "vst" method.

**4.2. Cell Clustering and Annotation**

To delineate cellular populations, dimensionality reduction was performed using principal component analysis (PCA) on the scaled gene expression matrix. The top 20 principal components were used to construct a shared nearest neighbor (SNN) graph, and cell clustering was carried out using the Louvain algorithm implemented in Seurat’s FindClusters function. Cellular identities were visualized using Uniform Manifold Approximation and Projection (UMAP). Cell type annotation was guided by the expression of well-established canonical marker genes for major hepatic and immune cell types. Specifically, B cells were identified by expression of CD19, CD79A, and MS4A1; T and NK cells by CD3D, CD8A, NKG7, GNLY, and GZMB; myeloid cells by CD14, LYZ, ITGAM, S100A8, and S100A9; endothelial cells by PECAM1, VWF, CDH5, and CLDN5; fibroblasts by DCN, COL1A1, ACTA2, and PDGFRB; and hepatocytes, including both normal and malignant populations, by expression of ALB, CYP3A4, APOA1, APOB, TF, and HP. Cluster-specific marker analysis was also performed to further refine annotations and validate classification accuracy across cellular subtypes.

**4.3. Copy Number Variation Analysis**

Malignant cells were identified using CopyKAT (v1.1.0), which infers copy number variation (CNV) from single-cell transcriptomic data to distinguish aneuploid malignant cells from diploid stromal or immune populations. Given that HCC 02 (the sole VETC-positive patient) showed high immune infiltration (T/NK cells: 67.3%; myeloid cells: 17.8%; hepatocytes: 7.7% of total cells), the default automatic reference selection of CopyKAT failed to identify any aneuploid hepatocytes in this sample, likely reflecting reference contamination by physiologically polyploid hepatocytes. To address this, CopyKAT was re-run with T/NK lymphocytes (n = 6,838) explicitly designated as the diploid normal reference population, as T/NK cells are lineage-diploid and contain no hepatocytic content. Under this pipeline, 362 of 785 hepatocytes (46.1%) in HCC 02 were classified as aneuploid. To validate these predictions independently of CNV inference, CopyKAT-aneuploid calls were cross-referenced with *GPC3* expression, a well-established HCC-specific marker that is not expressed in hepatocytes regardless of physiological ploidy state: 336 of 362 aneuploid cells (92.8%) were *GPC3*-positive, confirming malignant identity. As an empirical specificity check, the same revised pipeline was applied to non-neoplastic hepatocytes from the four VETC-negative patients and yielded 0% aneuploid classification (n = 8,615 cells), confirming that the analysis does not produce false-positive aneuploid calls in our dataset. Accordingly, 362 CopyKAT-confirmed malignant hepatocytes from HCC 02 were included in all downstream tumor cell analyses, yielding a final tumor cell pool of 12,247 cells across all five patients (HCC 01: 1,500; HCC 02: 362; HCC 03: 3,994; HCC 04: 4,168; HCC 05: 2,223).

**4.4. Gene Correlation Analysis**

To quantify VETC transcriptional activity at the single-cell level, a VETC score was computed for each tumor cell using the AddModuleScore function in Seurat (v5.1.0) with the 16 GeoMx-derived DEGs meeting log₂ fold change > 0.75 (*SIK1, MLXIPL, SERPINA4, BOK, DPP4, LAMTOR2, CRAT, ZNHIT1, MYORG, SLC66A2, ATXN7L1, GNAI1, CIART, IGFBP4, HDHD3, RXRA*; **Supplementary Table 4**). Cells were classified as VETC-high (top 33% VETC score, n = 4,083) or VETC-low (bottom 67%) based on the distribution across all 12,247 tumor cells. This continuous score-based classification was used in preference to cluster-based assignment, as it more directly captures cell-intrinsic VETC transcriptional activity independent of cluster resolution. Robustness of the gene set was assessed by comparing VETC scores computed at log₂ fold change thresholds of 0.5 (39 genes) and 0.75 (16 genes), which yielded highly correlated values (Pearson r = 0.910 across 12,247 tumor cells, **Supplementary Fig. 6a**). The Pearson correlation between VETC score and *LAMTOR2* expression was calculated at the single-cell level (n = 12,247 cells; unit of analysis = individual cell, not patient), and statistical significance was confirmed by a permutation test in which VETC score labels were randomly shuffled 5,000 times to generate a null distribution. To assess the consistency of the VETC–*LAMTOR2* association independently of inter-patient variability in baseline VETC scores, within-patient correlation analyses were performed separately for each patient, and within-patient VETC score tertiles (Low/Mid/High) were defined per patient with *LAMTOR2* expression compared across tertiles using Wilcoxon rank-sum tests. Group comparisons between VETC-high and VETC-low cells used Wilcoxon rank-sum tests.

**4.5. Pathway Analysis in Single-cell RNA-sequencing Data**

To investigate the functional pathways associated with *LAMTOR2* expression in VETC-positive HCC at single-cell resolution, differential expression analysis was performed between *LAMTOR2*-high and *LAMTOR2*-low cells within the VETC-high tumor clusters. Gene Ontology (GO) and Kyoto Encyclopedia of Genes and Genomes (KEGG) pathway enrichment analyses were conducted on the DEGs using the enrichGO and enrichKEGG functions in the clusterProfiler package (version 4.8.1). To characterize the biological direction of the GeoMx-derived DEGs at single-cell resolution, hallmark pathway scores (interferon-α response, interferon-γ response, IL6/JAK/STAT3 signaling, inflammatory response, Wnt/β-catenin signaling, angiogenesis, MSigDB v7.5.1) were computed for each tumor cell using AddModuleScore in Seurat (v5.1.0) and correlated with the single-cell VETC score using Pearson correlation. In parallel, pathway enrichment of the 58 GeoMx-derived downregulated DEGs (log₂ fold change < −0.75, p < 0.05; VETC-positive vs VETC-negative HCC) was assessed using enrichGO and enrichKEGG in the clusterProfiler package (v4.8.1) to characterize the biological processes associated with the downregulated transcriptional program in VETC-positive HCC.

**Supplementary Results**

**Analysis in The Cancer Genome Atlas database**

RNA expression of four genes (*LAMTOR2*, *DPP4*, *ZNHIT1* and *MLXIPL*) was analyzed in The Cancer Genome Atlas Liver Hepatocellular Carcinoma (TCGA-LIHC) cohort to assess the reliability of the GeoMx experiment results. Since VETC status annotation is not available in the TCGA dataset and the four genes were upregulated not only in VETC-positive HCC compared to VETC-negative HCC but also in HCC compared to non-neoplastic liver tissue, we compared tumor and normal tissues. The comparison between tumor and normal tissues from 49 HCC patients where matched normal tissue samples were available revealed that *LAMTOR2* RNA expression was significantly elevated in tumor tissues (p<0.001, **Supplementary Fig. 4a**). In contrast, *DPP4* and *MLXIPL* showed no significant differences in expression between tumor and normal tissues (p=0.953 and 0.836, respectively, **Supplementary Fig. 4b-c)**. Contrary to our GeoMx data, *ZNHIT1* expression was found to be decreased in tumor tissues compared to normal tissues (p=0.015, **Supplementary Fig. 4d**). Furthermore, among the 364 HCC cases where RNA expression data for all four genes were available, stratification based on the median *LAMTOR2* RNA expression revealed that *MLXIPL* and *ZNHIT1* expression were significantly elevated in the high *LAMTOR2* expression group (*MLXIPL*: 12.64 (12.03–13.13) vs. 13.12 (12.5–13.555), p<0.001; *ZNHIT1*: 11.45 (11.01–11.81) vs. 12.16 (11.64–12.69), p<0.001, **Supplementary Fig. 4e-g**).

**Single-cell RNA-sequencing characterization of *LAMTOR2* and the VETC transcriptional program**

Single-cell RNA-sequencing data from the five HCC patients comprised 49,229 cells, partitioned into seven major populations annotated by canonical marker genes: hepatocytes, tumor cells, T/NK cells, myeloid cells, B cells, endothelial cells, and fibroblasts (**Supplementary Fig. 5a**). *LAMTOR2* expression was highest in tumor cells and consistently elevated relative to non-neoplastic hepatocytes and stromal/immune populations (**Supplementary Fig. 5b-c**). CopyKAT-based copy number inference distinguished aneuploid (malignant) from diploid (non-malignant) populations, and the aneuploid compartment closely overlapped with the tumor cell annotation (**Supplementary Fig. 5d**). To refine intra-tumoral heterogeneity, tumor cells were re-embedded by Uniform Manifold Approximation and Projection (UMAP) after excluding non-malignant populations (**Supplementary Fig. 5f**), and a VETC transcriptional signature was constructed from the 16 GeoMx-derived DEGs at log₂ fold change > 0.75. Dot plot visualization of these 16 genes across tumor clusters confirmed cluster-level variability in their expression (**Supplementary Fig. 5e**), and the corresponding VETC scores projected onto the tumor UMAP delineated transcriptional patterns associated with the VETC phenotype (**Supplementary Fig. 5g**). Violin plot stratification by tumor cluster further revealed heterogeneity in VETC score distribution across tumor subpopulations (**Supplementary Fig. 5h**).

**Threshold robustness and validation of the *LAMTOR2*–VETC association at single-cell resolution**

Spatial transcriptomic differential expression between VETC-positive and VETC-negative HCC was re-analyzed using a unified log₂ fold change threshold of 0.75 (p < 0.05), identical to that applied in the single-cell RNA-sequencing analysis. To assess whether this stricter cutoff substantially altered the resulting transcriptional signature, VETC scores were independently computed for each tumor cell (n=12,247) using the 0.5 threshold gene set (39 genes) and the 0.75 threshold gene set (16 genes); the two scores were strongly concordant (Pearson r=0.910, **Supplementary Fig. 6a**). At the 0.75 threshold, 16 genes remained significantly upregulated and 58 genes significantly downregulated in VETC-positive HCC compared to VETC-negative HCC (**Supplementary Fig. 6b**). Among the 16 upregulated genes, four (*DPP4, LAMTOR2, MLXIPL,* and *ZNHIT1*) were also significantly upregulated when VETC-positive HCC was compared to non-neoplastic liver tissue, supporting the specificity of these candidates including *LAMTOR2* for the VETC-associated transcriptional program rather than for generic tumor signals.

To characterize the biological direction of the 58 GeoMx-derived downregulated DEGs (log₂ fold change < −0.75, p < 0.05, **Supplementary Table 5**), hallmark pathway scores were computed for each tumor cell and correlated with the single-cell VETC score. Interferon-α response, interferon-γ response, and IL-6/JAK/STAT3 signaling pathways showed positive correlation with low VETC score, whereas angiogenesis and Wnt/β-catenin signaling showed negative correlation (**Supplementary Fig. 6c**), indicating that the downregulated GeoMx DEGs predominantly reflect immune microenvironmental differences rather than tumor-intrinsic VETC features.

The association between *LAMTOR2* expression and the VETC transcriptional score was further validated at single-cell resolution. After incorporating 362 CopyKAT-confirmed malignant hepatocytes from HCC 02 (see Methods), the analysis included 12,247 tumor cells from all five patients. A permutation test in which VETC score labels were randomly shuffled 5,000 times generated a null distribution centered at zero, and the observed cell-level Pearson correlation (r=0.146) exceeded all 5,000 permuted values (empirical p<0.001, **Supplementary Fig. 6d**). Within-patient correlation analyses revealed a positive cell-level correlation in four of five patients (**Supplementary Fig. 6e**), and within-patient VETC score tertile comparisons which eliminate inter-patient baseline variability demonstrated significantly higher *LAMTOR2* expression in VETC-high than VETC-low tumor cells in the same four patients (HCC 02: p=0.008; HCC 03, HCC 04, and HCC 05: all p<0.001, **Supplementary Fig. 6f**). The single exception, HCC 01, had a documented history of cholangiocarcinoma and represents a biologically distinct case in which the hepatocyte-derived VETC program is not expected to recapitulate the same relationship with *LAMTOR2*.

Finally, stepwise validation of malignant hepatocyte identification in HCC 02, the sole VETC-positive patient in the scRNA-seq cohort, was performed to address the immune-dominated composition of this sample (T/NK cells: 67.3%; myeloid cells: 17.8%; hepatocytes: only 7.7% of total cells), which caused the default CopyKAT automatic reference selection to yield 0 aneuploid hepatocytes. Re-running CopyKAT with T/NK lymphocytes (n=6,838) explicitly designated as the diploid reference identified 362 of 785 hepatocytes (46.1%) as aneuploid. Of these, 336 (92.8%) were also *GPC3*-positive, independently corroborating their malignant identity. Application of the same revised pipeline to non-neoplastic hepatocytes of the four VETC-negative patients yielded 0% aneuploid classification (n=8,615 cells), confirming the specificity of these calls (**Supplementary Fig. 6g**).

**Supplementary Figures**

**Supplementary Fig. 1 Confirmation of vessels encapsulating tumor clusters (VETC)-positive cases by CD34 immunohistochemistry (IHC)**

**a** GeoMx Digital Spatial Profiling (DSP) images of the tumor microarray (TMA) slide of tumor tissue. Yellow circles highlight VETC-positive cases.

**b** CD34 IHC staining of the same TMA sections as shown in (**a**), with yellow circles indicating the VETC-positive cases.

**c** High magnification images of CD34 IHC staining from panel (**b**), with red circles indicating the VETC-positive regions. Case 017 (200x) is a VETC-negative case, while case 009 and 005 (400x) are VETC-positive.

**Supplementary Fig. 2 Representative immunohistochemistry (IHC) images of LAMTOR2 and CD34**

**a-d) LAMTOR2 IHC staining** (400x, scale bars: 100um, arrows indicate peripheral accentuation pattern)

**a** Weak cytoplasmic expression without peripheral accentuation. Cytoplasmic staining is diffuse within the cytoplasm with weak intensity.

**b** Weak peripheral accentuation pattern. Cytoplasmic staining is accentuated toward the membrane with weak intensity.

**c** Moderate cytoplasmic expression without peripheral accentuation. Staining intensity is increased in the cytoplasm compared to (**a**), but no accentuation toward the membrane noted.

**d** Moderate peripheral accentuation pattern. Staining intensity is increased compared to (**b**), with accentuation toward the membrane.

**e-f) CD34 IHCstaining** (200x, scale bars: 100um)

**e** VETC-negative case. CD34-positive endothelial cells show a capillary pattern without surrounding the tumor cells.

**f** VETC-positive case. CD34-positive endothelial cells completely surround the tumor cells.

**Supplementary Fig. 3**

**a** Heatmap showing the comparison of gene expression patterns among vessels encapsulating tumor clusters (VETC)-positive hepatocellular carcinoma (HCC), VETC-negative HCC, and non-neoplastic liver tissue

**b** Volcano plot comparing differentially expressed genes (DEGs) between VETC-positive and non-neoplastic liver regions

**c** Volcano plot comparing DEGs between VETC-negative and non-neoplastic liver regions

**d** The limit of quantitation (LOQ) value of four genes (blue: *MLXIPL*, green: *DPP4*, yellow: *ZNHIT1*, purple: *LAMTOR2*). All four genes exceeded the LOQ value in almost all areas of interest (*LAMTOR2*, 41/42; *DPP4* 37/42; *ZNHIT1* and *MLXIPL*, 42/42).

(VETC- HCC, vessels encapsulating tumor clusters-negative hepatocellular carcinoma; VETC+ HCC, vessels encapsulating tumor clusters-positive hepatocellular carcinoma)

**Supplementary Fig. 4 Analysis in The Cancer Genome Atlas (TCGA) database**

**a-d)** Boxplots of expression levels of key differentially expressed genes (*LAMTOR2*, *DPP4*, *MLXIPL,* and *ZNHIT1*) across tumor (n=49) and normal (n=49)

**a** *LAMTOR2* RNA expression was significantly elevated in tumor tissues (p<0.001).

**b** *DPP4* showed no significant differences in expression between tumor and normal tissues (p=0.953).

**c** *MLXIPL* showed no significant differences in expression between tumor and normal tissues (p=0.836).

**d** *ZNHIT1* expression was decreased in tumor tissues compared to normal tissues (p=0.015).

**e-g)** Boxplots of expression levels of *DPP4*, *MLXIPL,* and *ZNHIT1* across *LAMTOR2*-high group (n=183) and *LAMTOR2*-low group (n=181) based on the median *LAMTOR2* expression

**e** DPP4 expression showed no significant differences in expression between *LAMTOR2*-high group and *LAMTOR2*-low group.

**f** *MLXIPL* expression was significantly elevated in the *LAMTOR2*-high group.

**g** *ZNHIT1* expression was significantly elevated in the *LAMTOR2*-high group.

Statistical significance is indicated as *p < 0.05 and ****p < 0.0001.

**Supplementary Fig. 5**

**a** Major cell types identified from single-cell RNA-sequencing data of five hepatocellular carcinoma (HCC) patients. Cells were annotated based on canonical marker genes for B cells, T/NK cells, myeloid cells, endothelial cells, fibroblasts, hepatocytes, and tumor cells.

**b** Violin plots showing *LAMTOR2* expression across annotated cell types, with the highest expression observed in tumor cells

**c** Dot plots showing *LAMTOR2* expression across annotated cell types, with the highest expression observed in tumor cells

**d** CopyKAT-based classification distinguishes aneuploid (malignant) and diploid (non-malignant) cells within the dataset

**e** Dot plots show the expression of vessels encapsulating tumor clusters (VETC) signature genes derived from spatial transcriptomic differentially expressed genes (log₂ fold change > 0.75) across tumor clusters

**f** Uniform Manifold Approximation and Projection (UMAP) embedding of tumor cells only, showing refined clustering after excluding non-malignant populations

**g** Vessels encapsulating tumor clusters (VETC) scores calculated from spatial differentially expressed gene (DEG)-derived gene sets (log₂ fold change > 0.75) are visualized in tumor UMAPs, highlighting spatial transcriptional patterns associated with the VETC phenotype

**h** Violin plots display the distribution of VETC scores across tumor clusters, revealing heterogeneity in the VETC phenotype

**Supplementary Fig. 6**

**a** Scatter plot comparing vessels encapsulating tumor clusters (VETC) module scores in tumor cells (n = 12,247) calculated using two different log₂ fold change thresholds (> 0.5, 39 genes vs > 0.75, 16 genes) for defining the spatial transcriptomic-derived VETC signature, demonstrating that the two thresholds produce highly correlated scores (Pearson r = 0.91).

**b** Volcano plot of GeoMx differential expression between VETC-positive and VETC-negative hepatocellular carcinoma (HCC) (log₂ fold change > 0.75, p < 0.05), highlighting 16 upregulated genes (red) and 58 downregulated genes (blue; top genes with log₂FC < −1 labeled). The four genes *(DPP4, LAMTOR2, MLXIPL, ZNHIT1*) that are also significantly upregulated in VETC-positive HCC compared with non-neoplastic liver tissue (log₂FC > 1, p < 0.05) are highlighted in yellow.

**c** Bar plot showing Pearson correlation between the single-cell VETC score and hallmark pathway scores in tumor cells, colored by GeoMx direction. Interferon-α response, interferon-γ response, and IL-6/JAK/STAT3 signaling (down-regulated in VETC-positive GeoMx) show positive correlation with low VETC score, indicating that the downregulated differentially expressed genes (DEGs) reflect immune microenvironmental differences rather than tumor-intrinsic VETC markers, whereas angiogenesis and Wnt/β-catenin signaling (up-regulated in VETC-positive GeoMx) show negative correlation with low VETC score.

**d** Permutation test of the observed cell-level Pearson correlation between VETC score and *LAMTOR2* expression (all five patients, n = 12,247 tumor cells including 362 CopyKAT-confirmed malignant cells from HCC 02). VETC score labels were randomly shuffled 5,000 times to generate the null distribution; the observed r = 0.146 exceeded all 5,000 permuted values (empirical p < 0.001).

**e** Within-patient scatter plots of *LAMTOR2* expression versus VETC score across all five patients, with HCC 02 (VETC-positive) re-analyzed using CopyKAT-confirmed tumor cells (n = 362, aneuploid). The analysis unit is individual cells (n = 12,247), not patients, and a positive cell-level correlation is observed in four of five patients.
**f** Violin plots showing *LAMTOR2* expression stratified by within-patient VETC score tertile (Low, Mid, High) for each of the five patients. The within-patient tertile strategy removes inter-patient VETC score bias, and four of five patients show significantly higher *LAMTOR2* expression in VETC-high than VETC-low tumor cells (***p < 0.001, **p < 0.01, *p < 0.05, ns = not significant).
**g** Stepwise validation of malignant hepatocyte identification in HCC 02 (VETC-positive, n = 785 hepatocytes). Step 1: original CopyKAT with automatic reference selection yielded 0 aneuploid cells (0.0%), likely due to reference contamination in this immune-dominated sample. Step 2: re-running CopyKAT with T/NK cells designated as the explicit diploid reference identified 362 aneuploid hepatocytes (46.1%). Step 3: independent cross-validation by *GPC3* expression confirmed 336 of 362 cells (92.8%) as concordantly malignant, validating the CopyKAT calls with an independent lineage marker.

**Supplementary Table 1. Clinicopathological characteristics according to the presence of the vessels encapsulating tumor clusters pattern.**

|  | **Total**  **(n = 18)** | **VETC-negative**  **(n=11)** | **VETC-positive**  **(n=7)** | **p-value** | |
| --- | --- | --- | --- | --- | --- |
| **Clinical features** | | | | |  |
| Age (years) | 62 (56–66) | 62 (59–67) | 57 (54–64) | 0.364 | |
| Sex (male/female) | 17 (94%) / 1 (6%) | 11 (100%) / 0 (0%) | 6 (86%) / 1 (14%) | 0.389 | |
| Etiology |  |  |  | 0.683 | |
| HBV | 12 (66%) | 7 (64%) | 5 (72%) |  | |
| Alcohol | 3 (16%) | 3 (27%) | 0 (0%) |  | |
| MASLD | 1 (6%) | 0 (0%) | 1 (14%) |  | |
| HBV + alcohol | 1 (6%) | 1 (9%) | 0 (0%) |  | |
| Unknown | 1 (6%) | 0 (0%) | 1 (14%) |  | |
| Platelet (×10^9^/L) | 191 (140-226) | 213 (133-228) | 160 (145-196) | 0.659 | |
| PT-INR | 1.0 (1.0-1.1) | 1.0 (1.0-1.1) | 1.1 (1.0-1.1) | 1.000 | |
| Albumin (g/L) | 4.4 (4.3-4.6) | 4.4 (4.2-4.5) | 4.5 (4.3-4.6) | 0.142 | |
| Bilirubin (mg/dL) | 0.8 (0.6-1.1) | 0.6 (0.5-1.2) | 0.9 (0.6-0.9) | 0.648 | |
| AST (U/L) | 31 (22-39) | 38 (24-41) | 26 (18-31) | 0.057 | |
| ALT (U/L) | 26 (21-47) | 33 (25-53) | 17 (11-28) | 0.077 | |
| AFP (ng/mL) | 6.8 (2.6-16.4) | 7.5 (4.1-16.3) | 2.9 (2.3-60.9) | 0.724 | |
| PIVKA-II (mAU/mL) | 58 (41-156) | 49 (41-144) | 67 (37-170) | 1.000 | |
| **Pathological findings** | | | | |  |
| Satellite nodule | 1 (6%) | 1 (9%) | 0 (0%) | 1.000 | |
| Infiltrative gross type | 12 (67%) | 9 (82%) | 3 (43%) | 0.141 | |
| Tumor size (cm) | 3.0 (2.5–4.4) | 4.2 (2.8–4.7) | 2.6 (2.4–3.5) | 0.160 | |
| Subtype |  |  |  | 1.000 | |
| Conventional | 17 (94%) | 10 (91%) | 7 (100%) |  | |
| Steatohepatitic | 1 (6%) | 1 (9%) | 0 (0%) |  | |
| Worst Edmondson-Steiner grade III/IV | 13 (72%) | 8 (73%) | 5 (71%) | 1.000 | |
| Tumor necrosis | 6 (33%) | 4 (36%) | 2 (29%) | 1.000 | |
| Fatty change | 7 (39%) | 4 (36%) | 3 (43%) | 1.000 | |
| Hemorrhage/peliosis | 9 (50%) | 6 (55%) | 3 (43%) | 1.000 | |
| T stage |  |  |  | 0.872 | |
| T1a | 1 (6%) | 1 (9%) | 0 (0%) |  | |
| T1b | 7 (38%) | 3 (27%) | 4 (57%) |  | |
| T2 | 9 (50%) | 6 (55%) | 3 (43%) |  | |
| T3 | 1 (6%) | 1 (9%) | 0 (0%) |  | |
| Bile duct invasion | 1 (6%) | 0 (0%) | 1 (14%) | 0.389 | |
| Microvascular invasion | 9 (50%) | 6 (55%) | 3 (43%) | 1.000 | |
| Multiplicity | 5 (28%) | 4 (36%) | 1 (14%) | 0.596 | |
| **Background liver findings** | | | | |  |
| Underlying chronic hepatitis | 17 (94%) | 11 (100%) | 6 (86%) | 0.389 | |
| Lobular activity |  |  |  | 1.000 | |
| Absent | 6 (33%) | 3 (27%) | 3 (43%) |  | |
| Minimal | 10 (56%) | 6 (55%) | 4 (57%) |  | |
| Mild | 2 (11%) | 2 (18%) | 0 (0%) |  | |
| Portoperiportal activity |  |  |  | 1.000 | |
| Absent | 4 (22%) | 2 (18%) | 2 (29%) |  | |
| Minimal | 8 (45%) | 4 (37%) | 4 (57%) |  | |
| Mild | 4 (22%) | 3 (27%) | 1 (14%) |  | |
| Moderate | 2 (11%) | 2 (18%) | 0 (0%) |  | |
| Fibrosis |  |  |  | 0.563 | |
| Absent | 1 (5%) | 0 (0%) | 1 (14%) |  | |
| Portal | 2 (11%) | 1 (9%) | 1 (14%) |  | |
| Periportal | 3 (17%) | 2 (18%) | 1 (14%) |  | |
| Septal | 5 (28%) | 3 (27%) | 2 (29%) |  | |
| Cirrhosis | 7 (39%) | 5 (46%) | 2 (29%) | 0.637 | |
| Steatosis |  |  |  | 1.000 | |
| Absent | 0 (0%) | 0 (0%) | 0 (0%) |  | |
| Minimal (<5%) | 8 (44%) | 3 (27%) | 5 (71%) |  | |
| Mild (5-33%) | 10 (56%) | 8 (73%) | 2 (29%) |  | |
| **RNA expression** | | | | |  |
| *LAMTOR2* | 9.4 (6.0–11.5) | 6.6 (4.1–9.4) | 11.5 (10.6–15.2) | 0.011* | |
| *DPP4* | 3.3 (2.7-5.1) | 2.9 (2.5-3.4) | 6.7 (4.0-6.8) | 0.035* | |
| *ZNHIT1* | 16.6 (12.4-25.9) | 13.4 (11.8-18.7) | 26.2 (19.2-30.0) | 0.044* | |
| *MLXIPL* | 27.6 (18.7-34.4) | 18.9 (13.0-29.8) | 37.6 (30.6-44.4) | 0.008* | |

Note. Numbers are presented as medians (interquartile ranges) or values (percentages).

VETC, vessels encapsulating tumor clusters; HBV, hepatitis B virus; MASLD, metabolic dysfunction-associated steatotic liver disease; PT-INR, prothrombin time-international normalized ratio; AST, aspartate aminotransferase; ALT, alanine aminotransferase; AFP, alpha fetoprotein; PIVKA-II, prothrombin induced by vitamin K absence-II

**Supplementary Table 2. Clinicopathological characteristics of the validation cohort according to the presence of the vessels encapsulating tumor clusters pattern and peripheral accentuation pattern of LAMTOR2.**

|  | **Total**  **(n = 276)** | **VETC pattern** | |  | **LAMTOR2 peripheral accentuation** | |  |
| --- | --- | --- | --- | --- | --- | --- | --- |
|  |  | **Absent**  **(n=210)** | **Present**  **(n=66)** | **p-value** | **Absent**  **(n=211)** | **Present**  **(n=65)** | **p-value** |
| **Clinical features** |  |  |  |  |  |  |  |
| Age (years) | 58 (51–64) | 59 (52–65) | 57 (50–62) | 0.206 | 59 (52–65) | 56 (50–62) | 0.169 |
| Sex (male/female) | 228 (83%) / 48 (17%) | 176 (84%) / 34 (16%) | 52 (79%) / 14 (21%) | 0.452 | 174 (82%) / 37 (18%) | 54 (83%) / 11 (17%) | 1.000 |
| Underlying HBV | 229 (83%) | 177 (84%) | 52 (79%) | 0.396 | 175 (83%) | 54 (83%) | 1.000 |
| PT-INR | 1.09 (1.04–1.20) | 1.11 (1.04–1.21) | 1.07 (1.03–1.14) | 0.025* | 1.10 (1.04–1.21) | 1.09 (1.05–1.17) | 0.563 |
| Albumin (g/L) | 3.8 (3.3–4.2) | 3.8 (3.3–4.2) | 3.8 (3.4–4.1) | 0.858 | 3.8 (3.2–4.2) | 3.9 (3.5–4.2) | 0.202 |
| Bilirubin (mg/dL) | 1.0 (0.7–1.5) | 1.0 (0.7–1.6) | 0.9 (0.7–1.3) | 0.286 | 1.0 (0.7–1.8) | 1.0 (0.7–1.1) | 0.076 |
| AST (U/L) | 38 (29–61) | 38 (28–61) | 42 (31–58) | 0.641 | 38 (29–64) | 39 (28–51) | 0.262 |
| ALT (U/L) | 35 (25–57) | 35 (24–56) | 35 (26–61) | 0.560 | 36 (26–58) | 33 (22–48) | 0.169 |
| AFP (ng/mL) | 22 (6–429) | 17 (5–167) | 215 (10–3017) | 0.002* | 21 (6–288) | 27 (5–1352) | 0.471 |
| PIVKA-II (mAU/mL) | 97 (27–846) | 77 (26–634) | 333 (36–5670) | 0.007* | 99 (26–735) | 94 (31–1040) | 0.599 |
| **Pathological findings** |  |  |  |  |  |  |  |
| Infiltrative gross type | 159 (58%) | 119 (57%) | 40 (61%) | 0.673 | 118 (56%) | 41 (63%) | 0.381 |
| Tumor size (cm) | 4.0 (2.5–7.5) | 4.0 (2.3–7.0) | 5.5 (3.2–9.1) | 0.015* | 4.0 (2.5–7.9) | 4.0 (2.5–7.0) | 0.670 |
| Worst Edmondson-Steiner grade III/IV | 207 (75%) | 155 (74%) | 52 (79%) | 0.515 | 152 (72%) | 55 (83%) | 0.060 |
| T stage |  |  |  | 0.336 |  |  | 0.959 |
| T1a | 16 (6%) | 13 (6%) | 3 (5%) |  | 12 (6%) | 4 (6%) |  |
| T1b | 60 (22%) | 49 (23%) | 11 (17%) |  | 45 (21%) | 15 (23%) |  |
| T2 | 133 (48%) | 103 (49%) | 30 (45%) |  | 101 (48%) | 32 (49%) |  |
| T3 | 33 (12%) | 23 (11%) | 10 (15%) |  | 27 (13%) | 6 (9%) |  |
| T4 | 34 (12%) | 22 (11%) | 12 (18%) |  | 26 (12%) | 8 (13%) |  |
| Bile duct invasion | 18 (7%) | 11 (5%) | 7 (11%) | 0.213 | 15 (7%) | 3 (5%) | 0.772 |
| Major vessel invasion | 44 (16%) | 31 (15%) | 13 (20%) | 0.446 | 34 (16%) | 10 (15%) | 1.000 |
| Microvascular invasion | 116 (42%) | 77 (37%) | 39 (59%) | 0.002* | 86 (41%) | 30 (46%) | 0.531 |
| Multiplicity | 138 (50%) | 101 (48%) | 37 (56%) | 0.323 | 110 (52%) | 28 (43%) | 0.256 |
| MTM subtype | 23/176 (13%) | 12/120 (10%) | 11/56 (20%) | 0.094 | 17/129 (13%) | 6/47 (13%) | 1.000 |
| VETC pattern | 66 (23%) |  |  |  | 42 (20%) | 24 (37%) | 0.008* |
| **Background liver findings** |  |  |  |  |  |  |  |
| Lobular activity |  |  |  | 0.354 |  |  | 0.235 |
| Absent | 23 (8%) | 20 (10%) | 3 (5%) |  | 19 (9%) | 4 (6%) |  |
| Minimal | 91 (33%) | 64 (30%) | 27 (41%) |  | 63 (30%) | 28 (43%) |  |
| Mild | 138 (50%) | 106 (51%) | 32 (48%) |  | 111 (53%) | 27 (42%) |  |
| Moderate | 21 (8%) | 17 (8%) | 4 (6%) |  | 15 (7%) | 6 (9%) |  |
| Severe | 3 (1%) | 3 (1%) | 0 (0%) |  | 3 (1%) | 0 (0%) |  |
| Portoperiportal activity |  |  |  | 0.413 |  |  | 0.279 |
| Absent | 16 (6%) | 15 (7%) | 1 (2%) |  | 13 (6%) | 3 (5%) |  |
| Minimal | 62 (22%) | 46 (22%) | 16 (24%) |  | 46 (22%) | 16 (24%) |  |
| Mild | 137 (50%) | 105 (50%) | 32 (48%) |  | 100 (47%) | 37 (57%) |  |
| Moderate | 50 (18%) | 37 (18%) | 13 (20%) |  | 44 (21%) | 6 (9%) |  |
| Severe | 11 (4%) | 7 (3%) | 4 (6%) |  | 8 (4%) | 3 (5%) |  |
| Fibrosis |  |  |  | 0.769 |  |  | 0.825 |
| Absent/Portal | 45 (16%) | 34 (16%) | 11 (17%) |  | 33 (16%) | 12 (18%) |  |
| Periportal/Sepatal | 83 (30%) | 61 (29%) | 22 (33%) |  | 63 (30%) | 20 (31%) |  |
| Cirrhosis | 148 (54%) | 115 (55%) | 33 (50%) | 0.593 | 115 (54%) | 33 (51%) | 0.754 |
| Steatosis |  |  |  | 0.116 |  |  | 0.622 |
| Absent/Minimal (<5%) | 153 (55%) | 116 (55%) | 37 (56%) |  | 119 (56%) | 34 (52%) |  |
| Mild (5-33%) | 99 (36%) | 72 (34%) | 27 (40%) |  | 76 (36%) | 23 (35%) |  |
| Moderate (33-66%) | 22 (8%) | 21 (10%) | 1 (2%) |  | 15 (7%) | 7 (11%) |  |
| Severe (>66%) | 2 (1%) | 1 (1%) | 1 (2%) |  | 1 (1%) | 1 (2%) |  |
| **LAMTOR2 Immunohistochemistry** |  |  |  |  |  |  |  |
| Cytoplasmic expression with/without peripheral accentuation | 252 (91%) | 188 (90%) | 64 (97%) | 0.078 | 187 (89%) | 65 (100%) | 0.002* |
| Peripheral accentuation | 65 (24%) | 41 (19%) | 24 (36%) | 0.008* |  |  |  |
| **Prognostic findings** |  |  |  |  |  |  |  |
| Relapse | 169 (61%) | 121 (58%) | 48 (73%) | 0.040* | 120 (57%) | 49 (75%) | 0.011* |
| Death | 125 (45%) | 86 (41%) | 39 (59%) | 0.015* | 92 (44%) | 33 (51%) | 0.383 |

Note. Numbers are presented as medians (interquartile ranges) or values (percentages).

Abbreviations: VETC, vessels encapsulating tumor clusters; HBV, hepatitis B virus; PT-INR, prothrombin time-international normalized ratio; AST, aspartate aminotransferase; ALT, alanine aminotransferase; AFP, alpha fetoprotein; PIVKA-II, prothrombin induced by vitamin K absence-II; MTM, macrotrabecular massive.

**Supplementary Table 3. Characteristics of five human hepatocellular carcinoma patients of single cell RNA sequencing**

| Sample ID | Age | Gender | Biopsy date | Sample number | Etiology | HCC type | VETC |
| --- | --- | --- | --- | --- | --- | --- | --- |
| HCC 01 | 58 | Male | 2022-05-19 | 2 | HBV-LC | Recurred | Absent |
| HCC 02 | 72 | Male | 2023-07-24 | 3 | HBV-LC,  Alcoholic LC | Primary | Present |
| HCC 03 | 77 | Female | 2023-10-24 | 3 | HBV-LC | Primary | Absent |
| HCC 04 | 69 | Male | 2023-10-30 | 3 | HBV-LC | Recurred | Absent |
| HCC 05 | 83 | Female | 2023-11-03 | 2 | MASH | Primary | Absent |

Abbreviations: VETC, vessels encapsulating tumor clusters; HBV-LC, hepatitis B virus related liver cirrhosis; Alcoholic LC, Alcoholic liver cirrhosis; MASH, metabolic dysfunction-associated steatohepatitis

**Supplementary Table 4. Gene list of upregulated differentially expressed genes (log₂FC > 0.75, p < 0.05) in VETC-positive versus VETC-negative hepatocellular carcinoma in the spatial transcriptomic dataset**

| **Gene name** | **Log_2_ fold change** | **P-value** |
| --- | --- | --- |
| *SIK1* | 1.168443584 | 0.025998 |
| *MLXIPL* | 1.167522911 | 0.008754 |
| *SERPINA4* | 1.025752145 | 0.027883 |
| *BOK* | 0.977468 | 0.006476 |
| *DPP4* | 0.931074 | 0.035245 |
| *LAMTOR2* | 0.859399 | 0.024894 |
| *CRAT* | 0.799984 | 0.018995 |
| *ZNHIT1* | 0.794113 | 0.033474 |
| *MYORG* | 0.789833 | 0.034404 |
| *SLC66A2* | 0.787945 | 0.042998 |
| *ATXN7L1* | 0.766494 | 0.039152 |
| *GNAI1* | 0.765517 | 0.015554 |
| *CIART* | 0.756797 | 0.01226 |
| *IGFBP4* | 0.756499 | 0.049989 |
| *HDHD3* | 0.752514 | 0.03961 |
| *RXRA* | 0.751442 | 0.044995 |

**Supplementary Table 5. Gene list of downregulated differentially expressed genes (log₂FC < −0.75, p < 0.05) in VETC-positive versus VETC-negative hepatocellular carcinoma in the spatial transcriptomic dataset**

| **Gene name** | **Log_2_ fold change** | **P-value** |
| --- | --- | --- |
| *TSPY1* | -1.7736 | 0.02061 |
| *RBMY1J* | -1.4973 | 0.02288 |
| *H3C11* | -1.483 | 0.00746 |
| *H2BC3* | -1.2938 | 0.00812 |
| *CDY2B* | -1.2504 | 0.01164 |
| *H2BC8* | -1.2259 | 0.01314 |
| *H3C3* | -1.2062 | 0.01475 |
| *H3C4* | -1.1492 | 0.0189 |
| *H2AC12* | -1.1389 | 0.01097 |
| *H4C4* | -1.1092 | 0.01048 |
| *PAQR5* | -1.0839 | 0.04284 |
| *H1-1* | -1.0448 | 0.0359 |
| *H2AC11* | -1.0372 | 0.02484 |
| *KYNU* | -1.0264 | 0.03649 |
| *H3C10* | -1.0157 | 0.03774 |
| *DAZ4* | -1.0002 | 0.02439 |
| *NEB* | -0.9997 | 0.02077 |
| *H2BC7* | -0.9913 | 0.00986 |
| *DPYD* | -0.9904 | 0.00245 |
| *GABRE* | -0.9887 | 0.0244 |
| *H4C12* | -0.9864 | 0.01918 |
| *PRODH2* | -0.9619 | 0.04148 |
| *CCDC198* | -0.9543 | 0.02378 |
| *H2AC4* | -0.9509 | 0.00919 |
| *NLGN4Y* | -0.9419 | 0.0019 |
| *H2AC8* | -0.9373 | 0.02066 |
| *H2BC11* | -0.9301 | 0.04097 |
| *FAAP20* | -0.9112 | 0.00237 |
| *ALDH8A1* | -0.9 | 0.04283 |
| *AGRN* | -0.8922 | 0.02598 |
| *H2BC17* | -0.8903 | 0.02325 |
| *RCC1* | -0.8817 | 0.0237 |
| *RPL22* | -0.8765 | 0.01753 |
| *ULBP2* | -0.8619 | 0.0292 |
| *H3C1* | -0.8566 | 0.0251 |
| *RFPL4B* | -0.8554 | 0.00818 |
| *CELA3A* | -0.8316 | 0.04634 |
| *LY6E* | -0.8258 | 0.02665 |
| *ATCAY* | -0.8222 | 0.04457 |
| *SLC25A24* | -0.8143 | 0.00248 |
| *PRAMEF10* | -0.8106 | 0.04846 |
| *H1-4* | -0.8104 | 0.02774 |
| *CIDEB* | -0.8068 | 0.00886 |
| *IFI27L2* | -0.7952 | 0.00145 |
| *FZD6* | -0.7922 | 0.00654 |
| *RFPL1* | -0.7875 | 0.0215 |
| *LRRC28* | -0.7836 | 0.0067 |
| *LGALS1* | -0.7769 | 0.04058 |
| *ATAD3A* | -0.7718 | 0.01973 |
| *DDO* | -0.7711 | 0.00642 |
| *DVL1* | -0.7701 | 0.00126 |
| *TLE3* | -0.7592 | 0.0253 |
| *ASPM* | -0.7573 | 0.02551 |
| *HTR1D* | -0.7573 | 0.02002 |
| *ICMT* | -0.7537 | 0.01685 |
| *CLSPN* | -0.7526 | 0.01417 |
| *KIF2C* | -0.7512 | 0.03725 |
| *EPHA2* | -0.7506 | 0.02558 |
